# A Model of Silicon Dynamics in Rice: An Analysis of the Investment Efficiency of Si Transporters

**DOI:** 10.3389/fpls.2017.01187

**Published:** 2017-07-11

**Authors:** Gen Sakurai, Naoki Yamaji, Namiki Mitani-Ueno, Masayuki Yokozawa, Keisuke Ono, Jian Feng Ma

**Affiliations:** ^1^Statistical Modeling Unit, Division of Informatics and Inventory, Institute for Agro-Environmental Sciences, National Agriculture and Food Research Organization Tsukuba, Japan; ^2^Group of Plant Stress Physiology, Institute of Plant Science and Resources, Okayama University Kurashiki, Japan; ^3^Faculty of Human Sciences, Waseda University Tokorozawa, Japan; ^4^Crop-Climate Interaction Unit, Division of Climate Change, Institute for Agro-Environmental Sciences, National Agriculture and Food Research Organization Tsukuba, Japan

**Keywords:** silicon, mathematical model, silicon transporter, rice, silicon transport

## Abstract

Silicon is the second most abundant element in soils and is beneficial for plant growth. Although, the localizations and polarities of rice Si transporters have been elucidated, the mechanisms that control the expression of Si transporter genes and the functional reasons for controlling expression are not well-understood. We developed a new model that simulates the dynamics of Si in the whole plant in rice by considering Si transport in the roots, distribution at the nodes, and signaling substances controlling transporter gene expression. To investigate the functional reason for the diurnal variation of the expression level, we compared investment efficiencies (the amount of Si accumulated in the upper leaf divided by the total expression level of Si transporter genes) at different model settings. The model reproduced the gradual decrease and diurnal variation of the expression level of the transporter genes observed by previous experimental studies. The results of simulation experiments showed that a considerable reduction in the expression of Si transporter genes during the night increases investment efficiency. Our study suggests that rice has a system that maximizes the investment efficiency of Si uptake.

## Introduction

Once taken up by roots, mineral elements are transported to upper part with transpiration stream, followed by distributing to different organs and tissues. Understanding of the mechanisms that control the dynamics of both water and mineral elements is an important issue in plant science. Over the past decade, many transporters for uptake of mineral elements have been identified, including those in rice for N, P, K, Mg, B, Mn, Zn, Fe, and Si (Sasaki et al., 2016). These transporters are located in the plasma membrane. Moreover, some these transporters show polar localization. For example, Si influx transporter Lsi1 is localized to the distal side of the root exodermis and endodermis (Ma et al., 2006), while Si efflux transporter Lsi2 is localized to the proximal side of the same cells (Ma et al., 2007).

An important challenge is to develop a mathematical model that can simulate the dynamics of both water and mineral elements in the whole plant to quantitatively understand the complex mineral element transporting systems. Transporter expression depends on mineral concentrations in tissues (Sasaki et al., 2016). To quantify the dynamics of mineral element transport, it is necessary to consider multiple factors simultaneously including transporter activities in roots and shoots, the dynamics of xylem sap and phloem sap, and the expression levels of the transporter genes.

For water flow in plants, the models using an analogy with an electric circuit (Landsberg and Fowkes, 1978) is one of the most historical models. In these models, water potential (potential energy of water per unit volume relative to pure water) is treated like voltage in a circuit. Water flows according to the difference in water potentials. Because the flow of water in the phloem is strongly related to sucrose concentration, some models treat water and sucrose dynamics simultaneously (Daudet et al., 2002; Lacointe and Minchin, 2008; Lobet et al., 2014; Seki et al., 2015). For the transportation of substances in plant, several different concepts are used to model transport of mineral elements and organic pollutants from roots. In the compartment model, the plants are divided into several compartments (such as root and leaf), and substances are assumed to be transported among the compartments according to transition rates that are determined as parameter values (Fryer and Collins, 2003; Fantke et al., 2013; Trapp, 2014). However, these macro-scale models do not consider not only water flow dynamics but also such micro-scale characteristics as the location and activity of the transporters. On the other hand, in models that simulate the dynamics of substances at the micro-scale (Grieneisen et al., 2007, 2012; Sakurai et al., 2015; Yamaji et al., 2015; Foster and Miklavcic, 2016), substance flow is simulated by diffusion and convection in which the location and polarity of the transporters are considered. However, these models simulate dynamics at the cell or tissue level but not in the whole plant level.

The purpose of this study is to develop a new mathematical model that simulate silicon (Si) transport in whole plant in rice. Si is abundant in soils and is beneficial for plant growth (Ma and Takahashi, 2002). Si deposited in plant tissues enhances tolerance to abiotic and biotic stresses via alleviation of water stress, improvement of light interception characteristics by keeping the leaf blade erect, and an increase in resistance to diseases, pests, and lodging (Epstein, 1994; Savant et al., 1997; Ma and Takahashi, 2002). In rice roots, the passive transporter Lsi1 (OsLsi1) and the active transporter Lsi2 (OsLsi2) are involved in Si uptake (Ma et al., 2006, 2007). Both are located in the plasma membranes of the exodermal and endodermal cells, where Casparian strips are located. Lsi1 transports Si along the Si gradient between the plasma membrane, whereas Lsi2 transports Si from the symplast to the apoplast, and this transport is coupled with proton antiport (Ma et al., 2011). In rice nodes, Lsi2, Lsi3 (OsLsi3, active), and Lsi6 (OsLsi6, passive) transport Si from enlarged vascular bundles (EVB) to diffuse vascular bundles (DVB) for preferential distribution of Si to the grains (Yamaji and Ma, 2014; Yamaji et al., 2015). Dehydration stress decreases the expression of Lsi1 and Lsi2 via abscisic acid (ABA) in root (Yamaji and Ma, 2007). The expression of these transporter genes show a diurnal variation (Yamaji and Ma, 2007). More recently, it was reported that the expression of Lsi1 and Lsi2 genes is controlled by Si accumulation in the shoots, not in the roots (Mitani-Ueno et al., 2016). However, the mathematical models that simulate Si dynamics in whole plant have not been constructed yet.

In recent mathematical models of Si transport in both the root and node of rice (Sakurai et al., 2015; Yamaji et al., 2015), Si is assumed to be transported via diffusion and convection. In a previous study (Sakurai et al., 2015), we modeled the dynamics of Si transport from external solution to the cortex and stele of the root using a diffusion equation in which Si diffuses along the gradient of Si concentrations on a two-dimensional grid (4 × 4 μm). Using this micro-scale diffusion model, we could explain the dynamics of the micro-scale Si transport and could account for the characteristics of the spatial location of the transporters. The parameters of the models estimated using statistical computation methods, and the simulation output match the empirical data well. However, it is difficult to extend these models to whole-plant simulation.

In this study, we propose a new mathematical model that simulates Si transport in the whole plant using empirical data and knowledge from previous modeling studies. Using the model, we examined the factors and mechanisms affecting the expression of Si transporter genes. We assumed three possible signaling mechanisms that control the expression of Si transporter genes in this model: accumulation control, shortage control, and water stress control. Under accumulation control, expression is reduced by excess Si concentration in leaf cells. Under shortage control, expression is increased by low Si concentration in leaf cells. Under water stress control, expression responds to water stress (indicated by transpiration rate in this model). We compared the expression levels simulated by the models with those observed. Finally, using the model, we investigated the reason for the diurnal change of transporter gene expression levels in rice from the point of view of investment efficiency.

## Model and data

### Model structure

**Figure 1:**
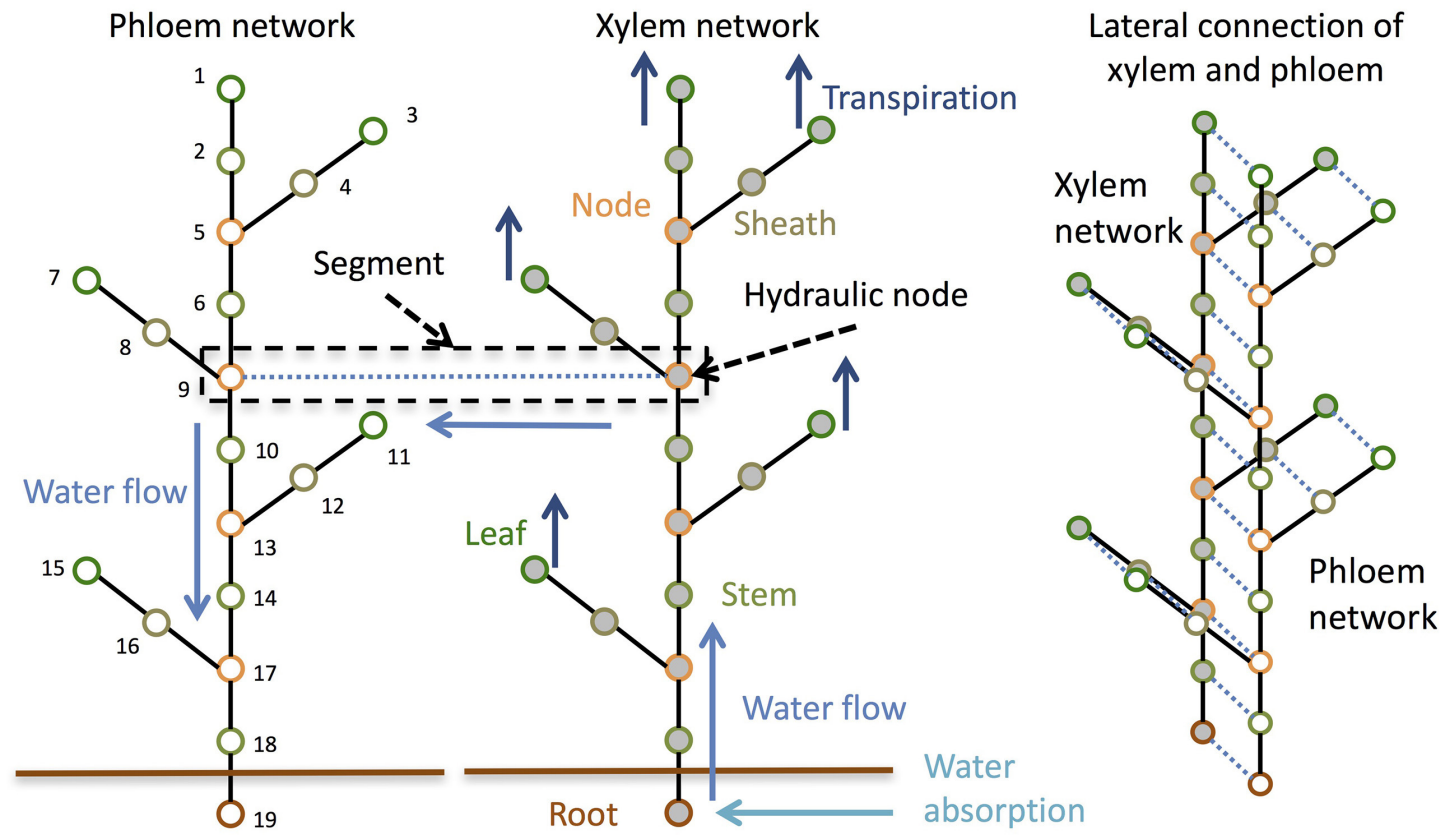
Schematic diagram of the model structure. The model is composed of a phloem network and a xylem network. The two networks are combined at each hydraulic node.

To consider the dynamics of water, sucrose, starch, Si, and the signals that control the expression level of Si transporter genes at the whole-plant level, we divided the whole plant into multiple points and connected them like in an electric circuit (Figure 1). In an electric circuit, a point of two or more elements is referred to as a “node,” but in biology the junction region of leaves and branches to the stem is also referred to as a “node;” therefore, we call a point in a circuit a “hydraulic node” (Figure 1).

### Water flow

The flow of water and sucrose in the xylem and phloem was calculated following the model proposed by Daudet et al. (2002). In this model, the axial water flow between hydraulic nodes conforms to the Ohm's law:

(1) $$J_{\text{W}\left(i,j\right)\text{X}}=-\frac{\Psi_{\text{X}\left(j\right)}-\Psi_{\text{X}\left(i\right)}}{r_{\text{X}\left(i,j\right)}}$$

where *J*_W(*i,j*)X_ is axial water flow in the xylem from the hydraulic node *i* to *j*, Ψ_X(*i*)_ and Ψ_X(*j*)_ is xylem water potential at the hydraulic node *i* and *j*, and *r*_X(*i,j*)_ is xylem flow resistance (Daudet et al., 2002). Water moves from one area to another in accordance with the gradient of water potential in the xylem (Cosgrove, 2010). Daudet et al. (2002) suggested a formula to describe the flow between the phloem and xylem and within the phloem. The lateral water flow between the xylem and phloem (*J*_W(*i*)Lat_) is described as:

(2) $$J_{\text{W}\left(i\right)\text{Lat}}=-\frac{\Psi_{\text{P}\left(i\right)}-\Psi_{\text{X}\left(i\right)}}{r_{\text{Lat}\left(i\right)}}$$

where Ψ_X(*i*)_ is water potential in the xylem, Ψ_P(*i*)_ is water potential in the phloem, and *r*_Lat(*i*)_ is the sum of the apoplastic pathway resistance between the xylem and phloem (Daudet et al., 2002). Axial water flow in the phloem [*J*_W(*i,j*)P_] is described as:

(3) $$J_{\text{W}\left(i,j\right)\text{P}}=-\frac{P_{\text{P}\left(j\right)}-P_{\text{P}\left(i\right)}}{r_{\text{P}\left(i,j\right)}}$$

where *P*_P(*i*)_ and *P*_P(*j*)_ is hydraulic (mainly turgor) pressure in the phloem at the hydraulic node *i* and *j* and *r*_P(*i,j*)_ is phloem flow resistance. Because gravity can be ignored when calculating water flow on a small scale, the following equation holds (Daudet et al., 2002):

(4) $$P_{\text{P}(i)}=\Psi_{P\left(i\right)}-\Pi_{i}$$

where Ψ_P(*i*)_ is water potential in the phloem and Π_*i*_ is osmotic potential. The latter can be described as:

(5) $$\Pi_{i}=-R\;\cdot \;T_{i}\;\cdot \;C_{\text{S}(i)}$$

where *R* is the universal gas constant, *T*_*i*_ is absolute temperature, and *C*_S(*i*)_ is sucrose concentration. The axial phloem solute flow [*J*_S(*i,j*)_] is described as:

(6) $$\left\{ \begin{array}{l} J_{\text{S}(i,j)}=J_{\text{W}(i,j)\text{P}}\;\cdot \;C_{\text{S}(i)},\;\text{when}\;J_{\text{W}(i,j)\text{P}}>0\\ J_{\text{S}(i,j)}=J_{\text{W}(i,j)\text{P}}\;\cdot \;C_{\text{S}(j)},\;\text{when}\;J_{\text{W}(i,j)\text{P}}<0 \end{array}\right.$$

where *J*_W(*i,j*)P_ is the axial water flow in the phloem. Because the purpose of the model developed in this study was to estimate the dynamics of mineral transport rather than sucrose flow, we ignored lateral sucrose flow for simplicity.

The flow of water should be conserved at any hydraulic node in the xylem and phloem. Therefore, the following equation should hold:

(7) $$\sum J_{\text{W}}=0$$

where *J*_W_ is the water flow from the target hydraulic node to the connected nodes. As this equation should hold at any node, we can estimate water potential at each hydraulic node at any time point by solving simultaneous equations under an appropriate boundary condition.

To calculate osmotic potential, we need sucrose concentration. In this model, we simply input the photosynthetic and transpiration rates as the boundary condition. Following photosynthesis, starch is synthesized in the leaf. The starch is dehydrated to sucrose and gradually loaded into the phloem, which is conveyed by the flow in the phloem, which follows the hydraulic pressure. The models of the dynamics of starch and sucrose in leaves and other tissues, which are similar to that of Daudet et al. (2002), are explained in Supplementary Information.

### Si transport

To understand the dynamics of Si in the whole shoot, we developed a simple two-compartment model that emulates the transport of Si in root. If we assume the compartmentation of the root cortex between the external solution (soil) and root stele, then the flow of Si from external solution (soil) to the cortex can be described as:

(8) $$J_{\text{M}\left(\text{o}:\text{c}\right)}=\alpha \;\cdot \;tr_{\text{exo}}\;\cdot \;C_{\text{M}:\text{out}}-p_{\text{cm}}\;\cdot \;\left(C_{\text{M}:\text{cor}}-C_{\text{M}:\text{out}}\right)$$

where *J*_M(o:c)_ is the flow of Si from external solution (soil) to the cortex, α regulates the expression level of the transporter (from 0 to 1), *tr*_exo_ is the transportation capability at the maximum expression level in exodermal cells, *C*_M:out_ and *C*_M:cor_ are Si concentrations in external solution and the cortex, respectively, and *p*_cm_ is the permeability of the cell membrane (Sakurai et al., 2015). Si is transported by endodermal transporters from the cortex to the stele; this flow can be described as:

(9) $$J_{\text{M}\left(\text{c}:\text{s}\right)}=\alpha \;\cdot \;tr_{\text{end}}\;\cdot \;C_{\text{M}:\text{cor}}-p_{\text{cm}}\;\cdot \;\left(C_{\text{M}\left(nr\right)}-C_{\text{M}:\text{cor}}\right)$$

where *J*_M(c:s)_ is the flow of Si from the cortex to the stele, *tr*_end_ is the transportation capability at the maximum expression level in the endodermis, *C*_M(*nr*)_ is the Si concentration in the stele (i.e., in the hydraulic node of the root), and *nr* is the sequential number of the hydraulic node of the root.

Note that both *tr*_exo_ (Equation 8) and *tr*_end_ (Equation 9) include the activity of both Lsi1 and Lsi2. The change of Si concentration can be described as:

(10) $$\frac{dC_{\text{M}:\text{cor}}}{dt}=\frac{J_{\text{M}\left(\text{o}:\text{c}\right)}-J_{\text{M}\left(\text{c}:\text{s}\right)}}{V_{\text{cor}}}$$

(11) $$\frac{dC_{\text{M}\left(nr\right)}}{dt}=\frac{J_{\text{M}\left(\text{c}:\text{s}\right)}-J_{\text{M}\left(nr,nr-1\right)}}{V_{\text{st}(i)}}$$

where *V*_cor_ is the tissue volume of the cortex (assumed to be 1 ml for simplicity), *J*_M(*nr*,*nr*−1)_ is the flow of Si from the root to the hydraulic node above the root, and *V*_st(*i*)_ is the tissue volume of the sieve tube.

We assumed that Si absorbed in the root is transported with the flow of water only in the xylem. Therefore, the following equation holds for any node:

(12) $$J_{\text{M}(i,j)}=C_{\text{M}(i)}\;\cdot \;J_{\text{W}(i,j)\text{X}}$$

where *J*_M(*i,j*)_ is the axial Si flow in the xylem and *C*_M(*i*)_ is Si concentration. The model assumes only transpiration as the force driving the water in the xylem; it does not consider the case when *J*_W(*i,j*)X_ is negative.

Using a diffusion equation for Si transport between EVB and DVB, we previously revealed the importance of the apoplastic barrier at the bundle sheath cells and suggested that transporters generate large differences in Si concentration between DVB and EVB to enable rice to transfer sufficient Si upward (Yamaji et al., 2015). Here, to model the dynamics of Si in the whole shoot, we simplified the model of Si distribution at the node as follows:

(13) $$J_{\text{M}\left(i,j\right)}=J_{\text{W}\left(i,j\right)}\;\cdot \;C_{\text{M}\left(i\right)\text{DVB}}$$

(14) $$J_{\text{M}\left(i,k\right)}=J_{\text{W}\left(i,k\right)}\;\cdot \;C_{\text{M}\left(i\right)\text{EVB}}$$

(15) $$C_{\text{M}\left(i\right)\text{DVB}}=\rho_{i}\;\cdot \;C_{\text{M}\left(i\right)\text{EVB}}$$

(16) $$C_{\text{M}\left(i\right)\text{EVB}}=\frac{J_{\text{W}\left(i,k\right)}+J_{\text{W}\left(i,j\right)}}{\rho_{i}\;\cdot \;J_{\text{W}\left(i,k\right)}+J_{\text{W}\left(i,j\right)}}\;\cdot \;C_{\text{M}\left(i\right)}$$

where *J*_M*(i,j)*_ and *J*_M*(i,k)*_ are Si flow, *J*_W*(i,j)*_ and *J*_W*(i,k)*_ are water flow, *C*_M_(*i*)_DVB_ and *C*_M(*i*)EVB_ are Si concentrations in DVB and EVB, respectively, and ρ_*i*_ determines how Si concentration increases in DVB. We assume that hydraulic node *j* is connected to hydraulic node *i* via DVB and that *k* is connected to *i* via EVB (see also Supplementary Figure 1). Increasing the concentration of Si in DVB can distribute a large amount of Si to upper developing tissues, to which DVB connects (Yamaji et al., 2015). This is the function of ρ_*i*_.

We assumed that Si is unloaded in each tissue according to the following equation:

(17) $$J_{\text{M}\left(i\right)\text{unload}}=k_{\text{M}:\text{unload}}\;\cdot \;C_{\text{M}\left(i\right)}\;\cdot \;V_{\text{con}\left(i\right)}$$

where *J*_M(*i*)unload_ is Si flow from the xylem to tissue cells, *k*_M:unload_ is a parameter, and *V*_con(*i*)_ is the tissue volume of the conduit. The Si concentration in tissue cells, *C*_M(*i*)cyt_, is calculated as:

(18) $$\frac{dC_{\text{M}\left(i\right)\text{cyt}}}{dt}=\frac{J_{\text{M}\left(i\right)\text{unload}}}{V_{\text{cyt}(i)}}$$

where *V*_cyt(*i*)_ is the volume of the cytoplasm in the tissue.

### Transport of a signaling substances

Lsi2 expression is decreased by high Si accumulation in the shoot through an unknown signal from shoots to roots (Yamaji and Ma, 2011), and the expression of Lsi1 and Lsi2 is decreased by dehydration stress (Yamaji and Ma, 2007, 2011); the response to dehydration stress is more rapid than the response to Si accumulation. In this study, we used the following models to investigate the dynamics of the unknown signal. We considered the following three types of models for generation of the signal:

(19) $$\text{Accumulation control}:GR=slp_{\text{c}}\;\cdot \;C_{\text{M}\left(i\right)\text{cyt}}$$

(20) $$\text{Shortage control}:GR=slp_{\text{r}}\;\cdot \;(1-C_{\text{M}\left(i\right)\text{cyt}})$$

(21) $$\text{Water stress control}:GR=slp_{\text{J}}\;\cdot \;Trans$$

where *GR* is the rate of signal generation, *slp*_c_, *slp*_J_, and *slp*_r_ are parameters that determine the generation rate of signal in response to the target factor, *C*_M(*i*)cyt_ is Si concentration in leaf cells, and *Trans* is transpiration rate in leaf. For simplicity, we assumed that the signal is generated only in the leaf. Moreover, because the signal is an unknown substance, we did not define its units. Equation (19) represents that the signal is generated according to Si concentration in leaf cells. Equation (20) represents that the signal is generated according to the transpiration rate in leaf. In this situation, we assume that the water stress is in proportion to the transpiration rate. This assumption may be rough approximation because the water stress in a plant would be affected by several factors such as soil moisture, water absorption history, and transpiration rate. However, we adopt this simple assumption because estimating exact water stress is beyond the purpose of this study. Equation (21) represents that the signal is generated according to the shortage of Si in leaf cells. In this situation, the signal transmits the information about shortage of Si. On the other hand, in Equations (19), (20), the signal transmits the information about excess of Si. The signal decays according to decay rate *dec*. That is,

(22) $$\frac{dC_{R\left(i\right)}}{dt}=-dec\;\cdot \;C_{R\left(i\right)}+GR$$

where *C*_R*(i*)_ is signal concentration. We assumed that the generated signal is transferred via the phloem water flow. When the signal reaches the root, the expression level of the Si transporter is regulated according to the signal concentration as follows:

(23) $$\alpha \;=\text{ max}(0,-C_{\text{R}\left(nr,\;t-\chi \right)}+1)$$

(24) $$\alpha \;=\text{ min}(1,C_{\text{R}\left(nr,\;t-\chi \right)})$$

where α is a factor that regulates transporter expression level, *C*_R*(nr,t*)_ is signal concentration at time *t* (hour), and χ is delay of time. It is set to 0 for Accumulation control assumption and Shortage control assumptions but set to 5 for Water stress control situation because the previous study suggests that there is time lag until the decrease of the expression level by dehydration stress (Yamaji and Ma, 2011). Equation (23) is used for Accumulation control and Water stress control assumptions. Equation (24) is used for Shortage control assumption.

### Simulation settings

**Figure 2:**
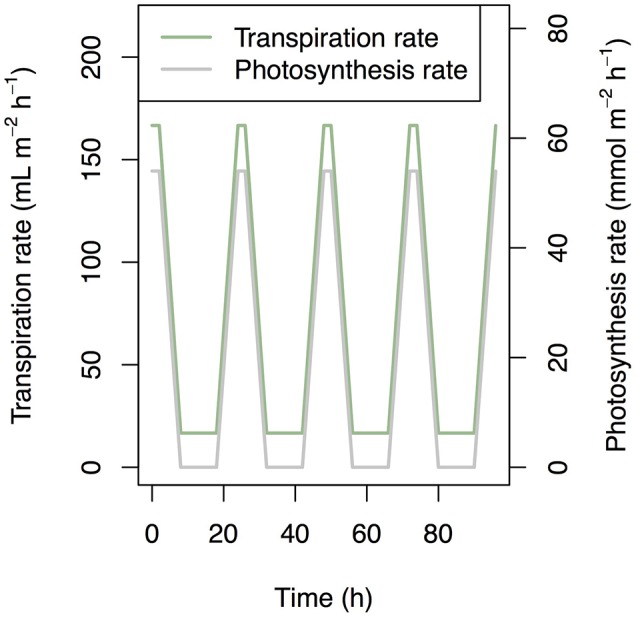
Time series of transpiration rate and photosynthesis rate that was used as a boundary condition in simulation.

We set the values of transpiration rate, photosynthesis rate, and temperature as input data. We set the standard transpiration rate to 0.4 ml cm^−2^ day^−1^ (Kuwagata et al., 2012) and standard photosynthesis rate to 0.0015 mol cm^−2^ s^−1^. We assumed (i) 10-h night, (ii) 2-h peaks of photosynthesis and transpiration (Figure 2), and (iii) a transpiration rate during darkness of 10% of the standard transpiration rate. We also assumed a simplified rice plant structure: (i) the root represented by one segment; (ii) five leaves; (iii) identical internode length; (iv) identical sheath length; and (vi) constant biomass during simulation. The other structural settings are described in Supplementary Table 1. The simulation period was 4 days.

### Parameters for transporters in roots

To estimate the values of *tr*_exo_, *tr*_end_, and *p*_cm_, we used time-series Si concentrations in xylem sap of 1-month-old seedlings exposed to 1.0 mM Si solution and measured every 5 min (Sakurai et al., 2015). We estimated parameter distribution using Markov chain Monte Carlo methods with the following likelihood function:

(25) $$L\left(\theta |Data\right)=\prod \frac{1}{\sqrt{2\pi \sigma_{t}}}\exp\left(-\frac{(C_{M(nr),t}(\theta )-C^{\prime }{\;}_{M\left(nr\right),\;t}){\;}^{2}}{2\sigma_{t}^{2}}\right)$$

where *L* is the likelihood of parameter set θ with the observed data set *Data*, σ_*t*_ is the standard deviation of the error distribution at time *t, C*_*M*(*nr*),*t*_(θ) is the Si concentration in xylem sap at time *t* estimated with the parameter set θ, and *C*′_*M*(*nr*),*t*_ is the observed Si concentration. We assumed that σ_*t*_ is equivalent to the standard deviation of the observed data. The initial setting for *C*_M:cor_ (Equation 8) was 0.0 mM. The observed time-series data for xylem sap and estimated data are shown in Supplementary Figure 2. The model adequately estimated the observed Si concentrations.

### Estimation of parameters relevant to respiration

We estimated sucrose dynamics under several parameter settings. The estimated parameter values were 0.1, 0.15, and 0.2 for k4 and 2.0e-5, 4.0e-5, 6.0e-5, 8.0e-5, 10.0e-5, 12.0e-5, 14.0e-5, 16.0e-5, 18.0e-5, and 20.0e-5 for k1 (see Supplementary Information for k1 and k4). The parameter set where sucrose has the stable cyclic dynamics (k1 = 8.0e-5 and k4 = 0.1) was used for the simulation experiment (Supplementary Figure 3).

### Simulation of Si dynamics

To estimate Si dynamics, we used Equations (19), (20), or (21). In each case, we simulated Si dynamics with several parameter sets for *slp* and *dec*. For the other parameters and input data, we used the same values in all equations. The definitions of variables are described in Supplementary Table 2. The parameter values used for the simulation are described in Supplementary Tables 2, 3.

### Investment efficiency

To evaluate investment efficiency, we defined it as:

(26) $$IE=\frac{\int_{t=stri}^{t=endi}J_{\text{M}\left(1,t\right)\text{unload}}\;dt\;}{\int_{t=stri}^{t=endi}\alpha_{t}\;dt\;}$$

where *IE* is investment efficiency, *t* is time, *stri* is the starting time of the calculation, *endi* is the ending time of the calculation, *J*_M(*i,t*)unload_ is Si flow from the xylem to the cells of top leaf at time *t*, and α_*t*_ is a factor that regulates transporter expression level at time *t*. This equation means that the investment efficiency is the amount of Si accumulated in top leaf divided by the total expression level of Si transporter genes.

### Simulation under natural environment

Finally, we simulated Si dynamics under the natural environmental condition. In the above simulation setting, the artificial pattern of transpiration rate and photosynthesis rate were used (Figure 2). To confirm the result that is found in the above simulation experiment, we simulated the Si dynamics with the input data that was measured in the field experiment. The data were observed at the paddy site using the eddy covariance method during the growing season in 2004. The observation site was located at Mase, Tsukuba City, Ibaraki prefecture, Japan. In this observation, not only LE (Latent heat flux) and NEE (Net Ecosystem CO_2_ Exchange) but also LAI (Leaf Area Index) were measured. We used LE and NEE for the estimation of transpiration and photosynthesis rates, respectively, assuming that the effects of evaporation from the water surface and heterotrophic respiration from the soil on the observed fluxes were negligible in our analysis. We used the data during day 33 and 35 after transplanting.

## Results

### Si dynamics and expression of Si transporter genes under accumulation control

**Figure 3:**
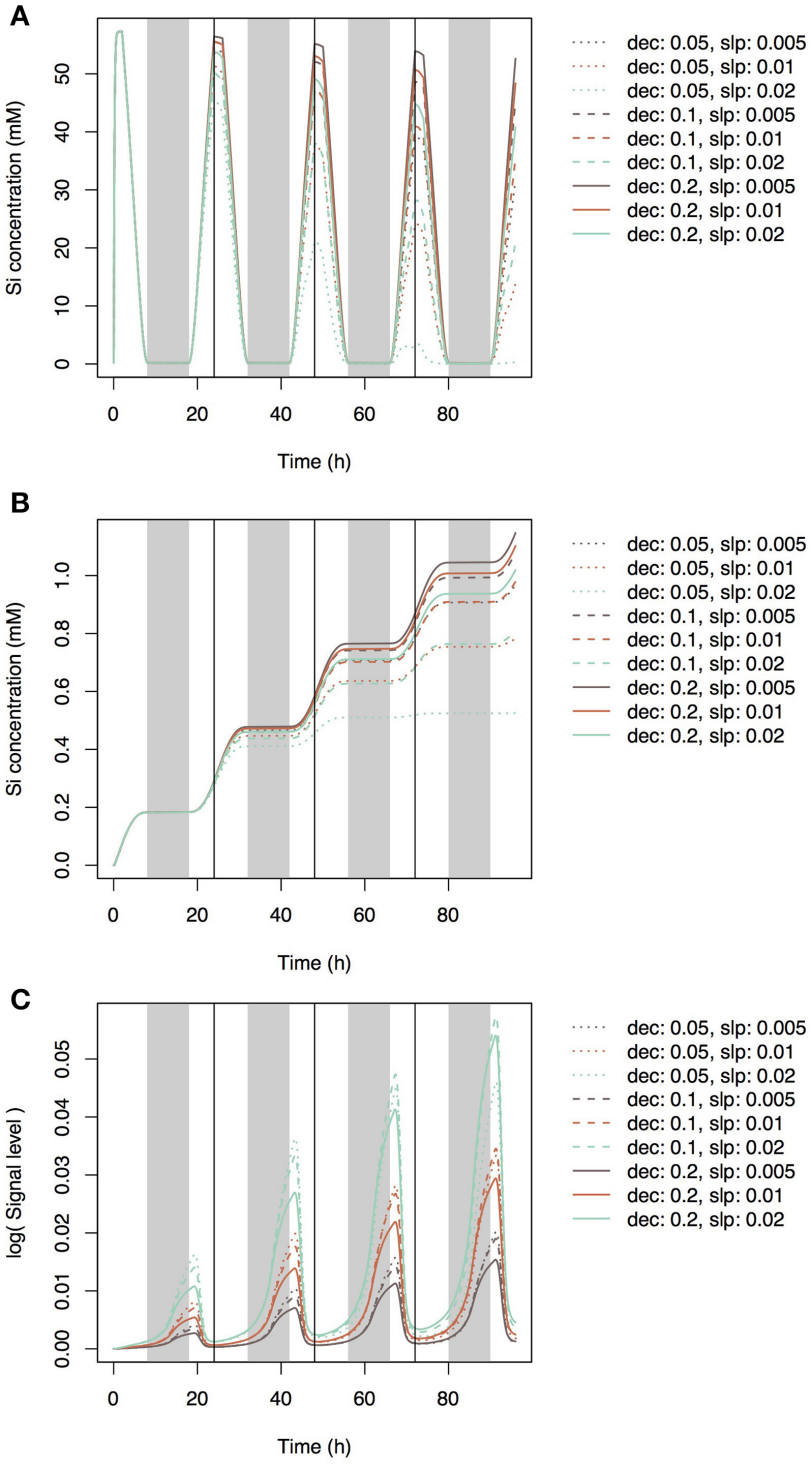
Si concentration in xylem sap **(A)** and leaf cells **(B)** of the top leaf and the signal level in xylem sap **(C)** of the top leaf simulated with multiple parameter sets under the accumulation control assumption. Parameter *dec* is the decay rate of the signaling substance. Parameter *slp* is the generation rate of the signaling substance and corresponds to *slp*_c_ in Supplementary Table 2.

**Figure 4:**
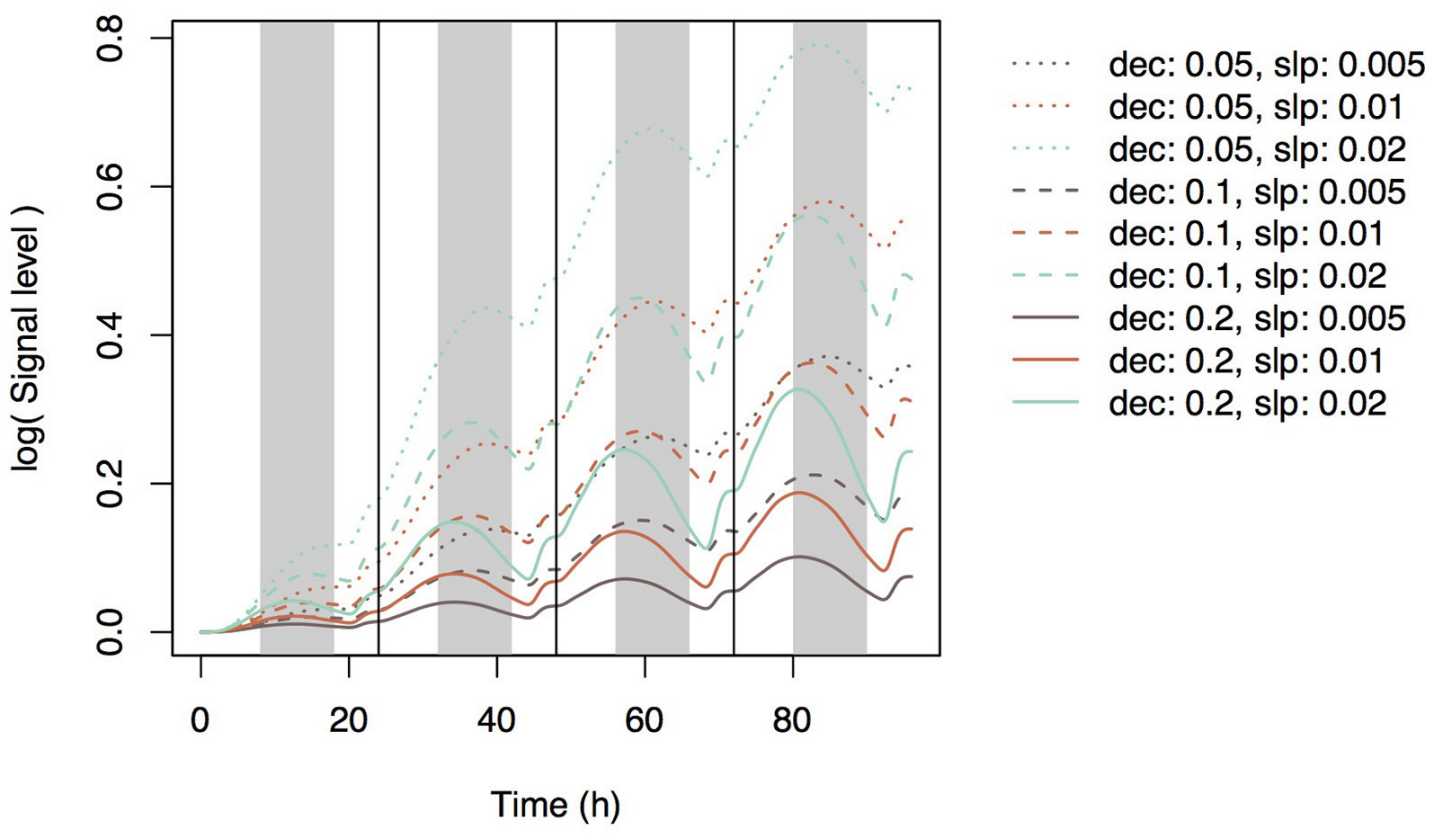
Signal level in xylem sap of the root simulated with multiple parameter sets. Parameter *dec* is the decay rate of the signaling substance. Parameter *slp* is the generation rate of the signaling substance and corresponds to *slp*_c_ in Supplementary Table 2.

**Figure 5:**
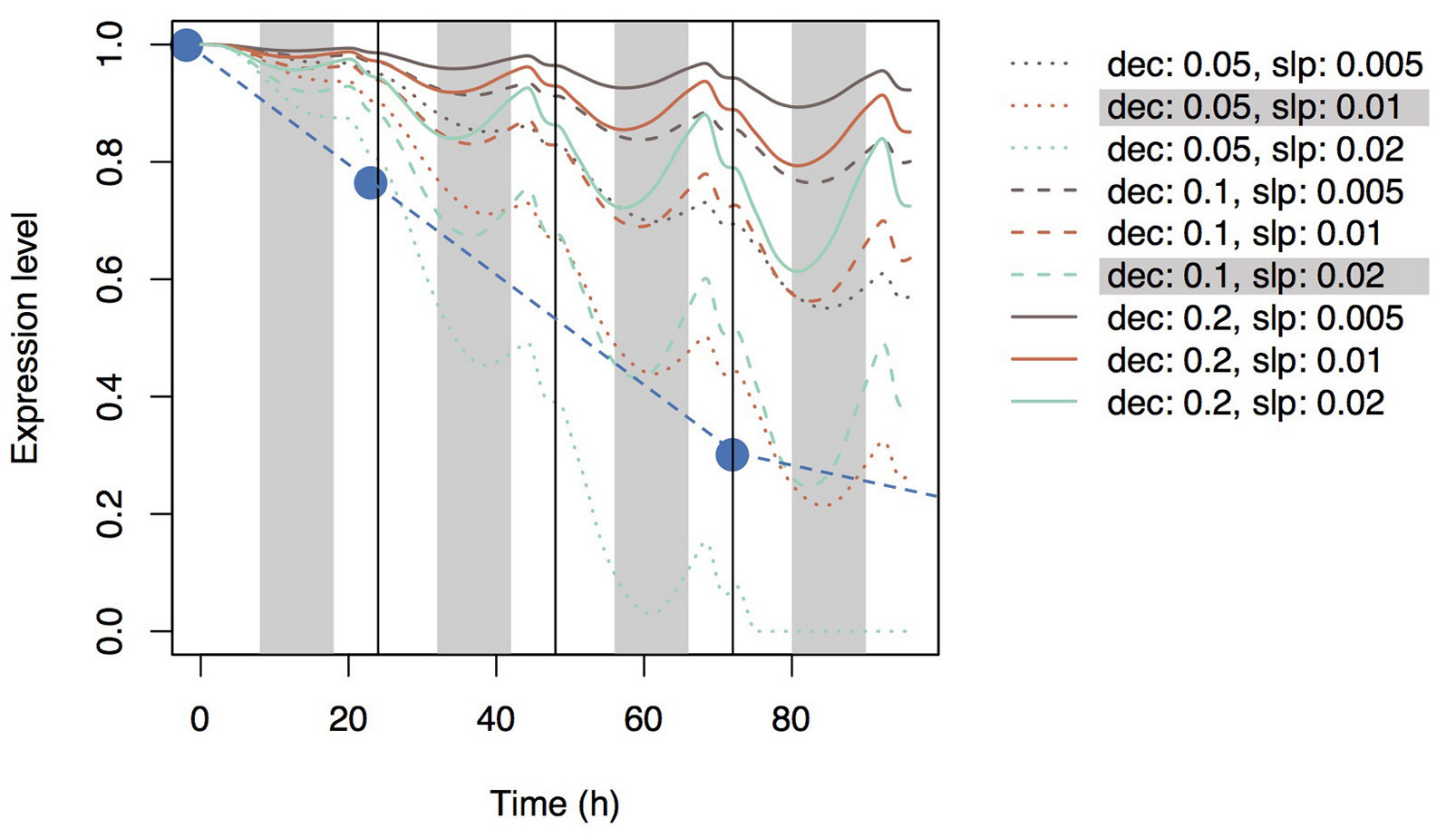
Time series of the expression levels of Si transporter genes simulated with multiple parameter sets under the accumulation control assumption. Parameter *dec* is the decay rate of the signaling substance. Parameter *slp* is the generation rate of the signaling substance and corresponds to *slp*_c_ in Supplementary Table 2. Blue circles indicate the observed values of the mean expression level of *Lsi*1 (Yamaji and Ma, 2011).

Using Equation (19), we simulated the model with several parameter sets (0.05, 0.1, and 0.2 for *dec* and 0.005, 0.01, and 0.02 for *slp*_c_). During the day, Si concentration in the xylem of the top leaf rapidly increased and then decreased (Figure 3A) according to changes in leaf transpiration rate (Figure 2). At night, Si concentration reached zero, and then it increased again according to the increase in transpiration rate. Although Si concentration did not reach zero in the lower leaf (Supplementary Figure 4), a similar pattern was observed. Though the pattern was similar at all parameter settings, Si concentrations during the day were low in the models with low *dec* values (slow decay of signaling substance) and high *slp*_c_ values (rapid generation of the signaling substance). Si concentration in leaf cells gradually increased, and the differences among parameter sets became apparent after 24 h (Figure 3B). Si concentration in leaf cells was the lowest at *dec* = 0.05, *slp*_c_ = 0.02, and was only about half of that at *dec* = 0.2, *slp*_c_ = 0.005. The pattern was the same in the lowest leaf (Supplementary Figure 5). The signal level in phloem sap of the top leaf (Figure 3C) and roots (Figure 4) gradually increased with time and reached local maxima at dawn for the top leaf and at dusk for the roots. The signal level was the lowest at *dec* = 0.2, *slp*_c_ = 0.005, and was about one tenth of that at *dec* = 0.05, *slp*_c_ = 0.02) for the roots. The expression level of the transporter genes in roots gradually decreased with time (Figure 5). Two parameter sets (*dec* = 0.05, *slp*_c_ = 0.01; *dec* = 0.1, *slp*_c_ = 0.02) fit best the observed expression levels (mean mRNA levels) of Lsi1 measured using real-time RT-PCR by Yamaji and Ma (2011). Local maxima were reached during the day with all parameter sets.

### Expression of Si transporter genes under shortage control

**Figure 6:**
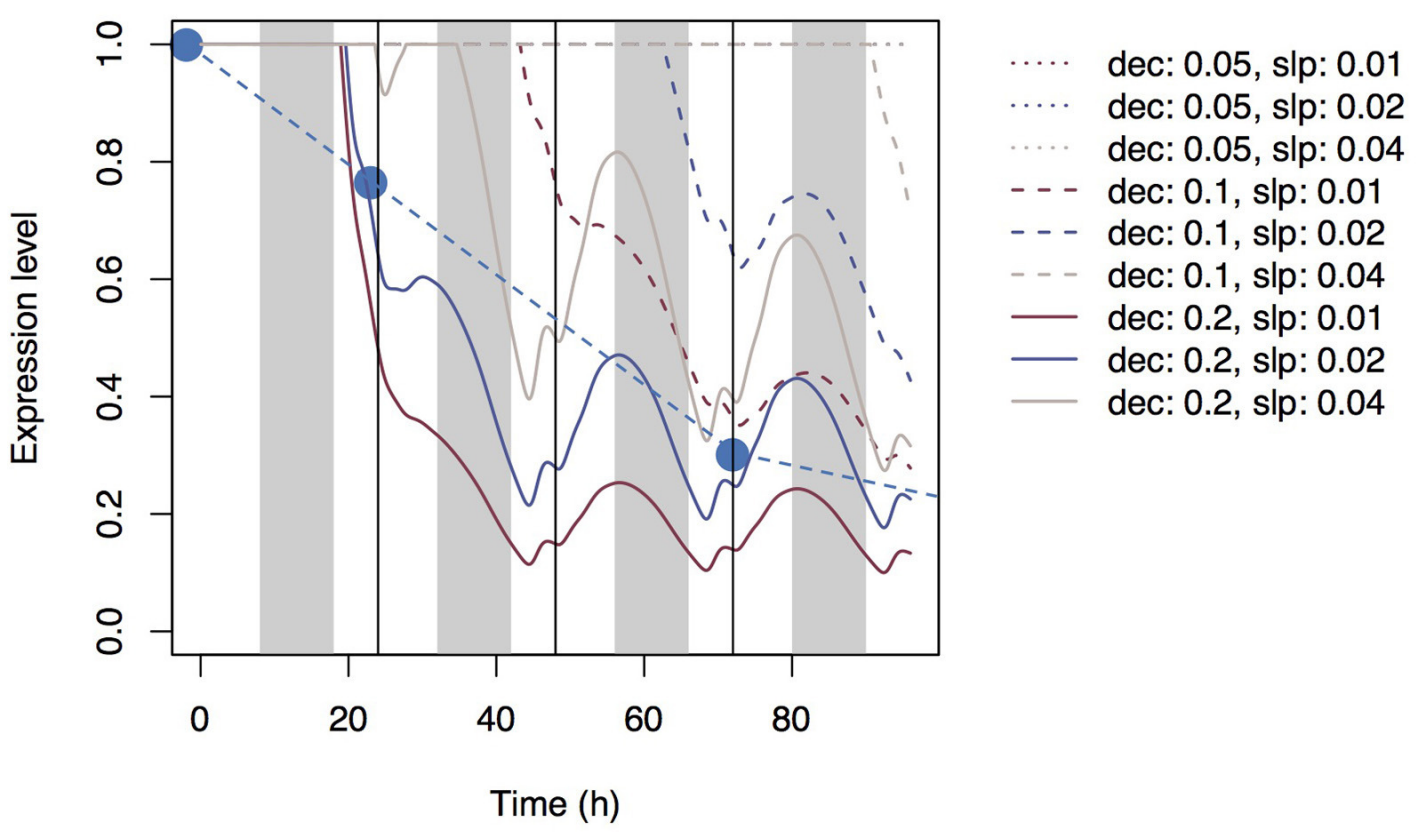
Time series of the expression levels of Si transporter genes simulated with multiple parameter sets under the shortage control assumption. Parameter *dec* is the decay rate of the signaling substance. Parameter *slp* is the generation rate of the signaling substance and corresponds to *slp*_r_ in Supplementary Table 2. Blue circles indicate the observed values of the mean expression level of *Lsi*1 (Yamaji and Ma, 2011).

Using Equation (20) and the same parameter sets, we simulated the expression level of the transporter genes in roots (Figure 6). As with accumulation control, the expression level gradually decreased with time, but local maxima were reached at dusk and local minima were reached at dawn with all parameter sets.

### Si dynamics under water stress control

**Figure 7:**
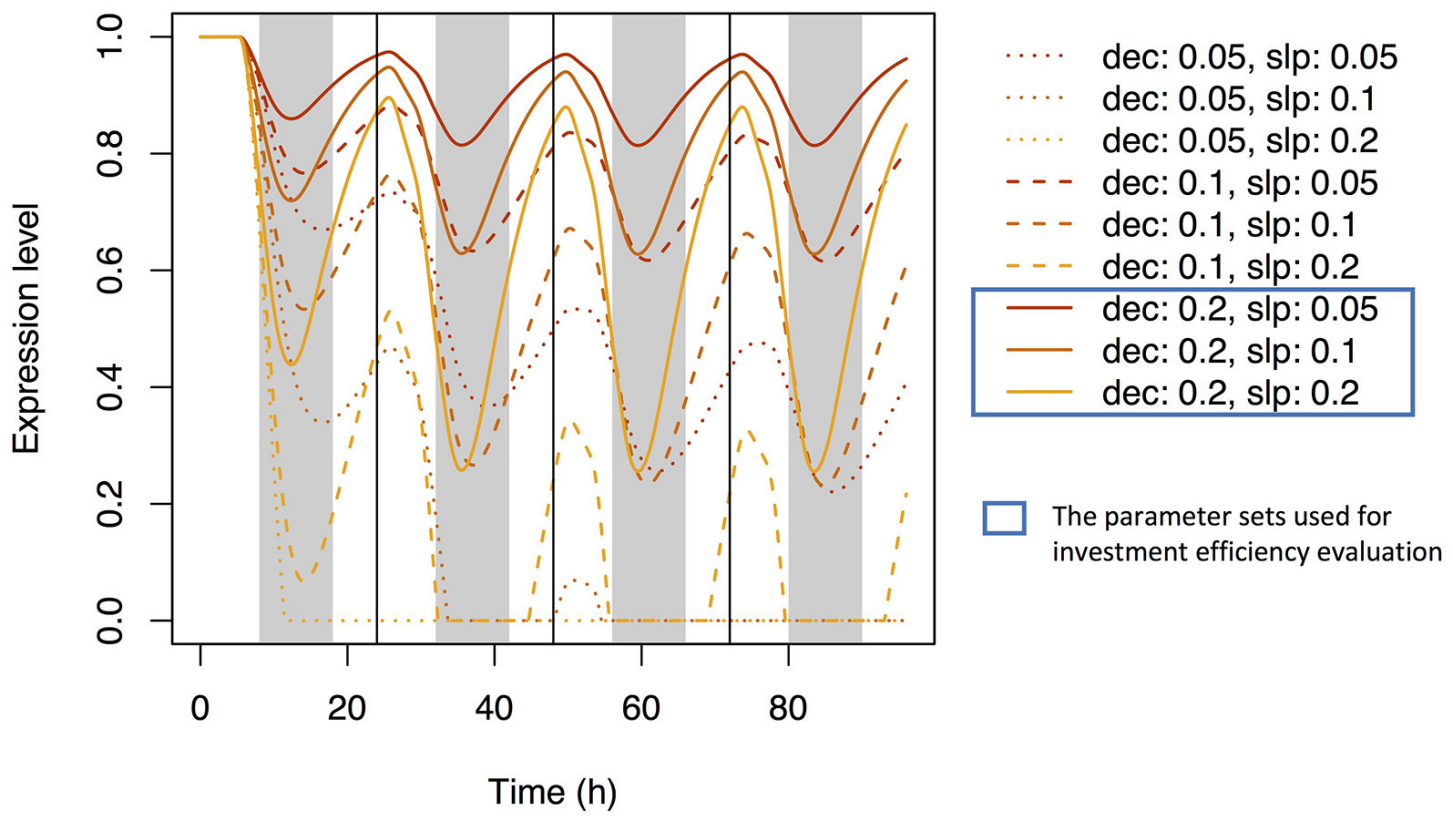
Time series of the expression level of Si transporter genes simulated with multiple parameter sets under the water stress control assumption. Parameter *dec* is the decay rate of the signaling substance. Parameter *slp* is the generation rate of the signaling substance and corresponds to *slp*_J_ in Supplementary Table 2.

Using Equation (21) and the same parameter sets, we simulated the dynamics of Si concentration in the xylem (Supplementary Figure 6A) and in top-leaf cells (Supplementary Figure 6B). The patterns were similar to those under accumulation control. However, the pattern of the signal level in xylem sap differed: the local maxima were reached at dusk, but the signal level did not increase with time (Supplementary Figure 6C). A diurnal expression pattern in roots was found with some parameter sets (Figure 7); local maxima were reached during the day and local minima at night. This pattern is similar to that observed by Yamaji and Ma (2007) for the *Lsi1* expression level in the root.

### Investment efficiency

**Figure 8:**
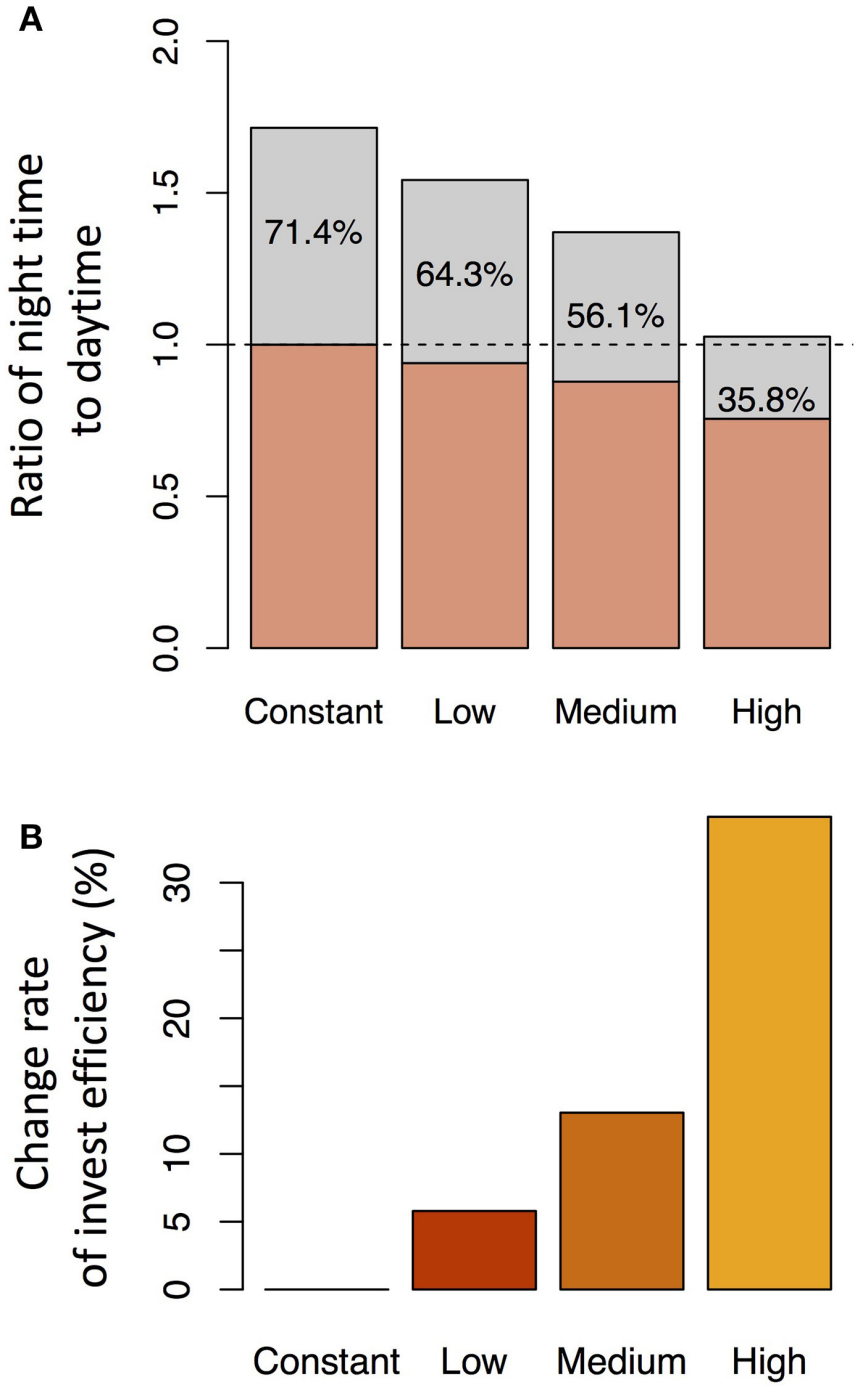
Ratio of the investment during the night to that during the day for each parameter set **(A)** and the change rate of the investment efficiency relative to that under the constant expression of the transporter genes **(B)**. The parameter sets were constant (no sensitivity of Si transporter expression to water stress; *dec* = 0.0, *slp*_J_ = 0.0), low sensitivity (*dec* = 0.2, *slp*_J_ = 0.05), intermediate sensitivity (*dec* = 0.2, *slp*_J_ = 0.1), and high sensitivity (*dec* = 0.2, *slp*_J_ = 0.2) under the water stress control assumption (see Figure 7).

We investigated the reason why the expression level of the transporter genes shows diurnal variation in the point of view of investment efficiency. We used *dec* = 0.2 with *slp*_J_ = 0.05 (low sensitivity of Si transporter expression to water stress), 0.1 (intermediate), or 0.2 (high) under the water stress control assumption (see Figure 7). Under “constant” setting (which means that the expression level of the transporter does not change in response to water stress), the investment during the night was 71.4% of that during the day. The nighttime investment decreased with increasing sensitivity (Figure 8A). As the results, the investment efficiency increased with increasing sensitivity: it was 5.8% at low sensitivity, 13.0% at intermediate sensitivity, and 34.9% at high sensitivity (Figure 8B).

### Simulation under natural environment

**Figure 9:**
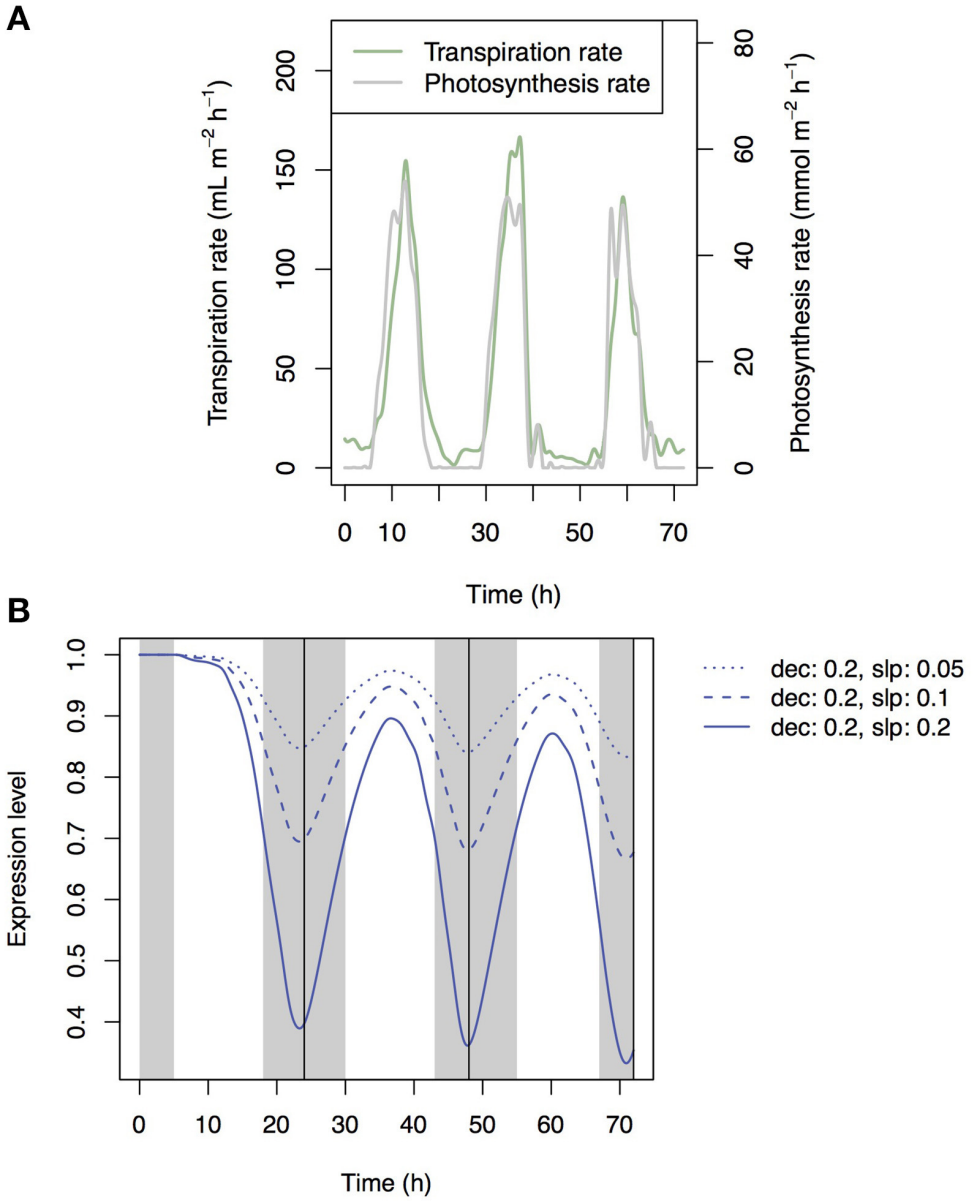
Time-series of measured transpiration and photosynthesis rates **(A)** and of the transporter expression level at low, intermediate, and high sensitivity simulated under the water stress control assumption **(B)**.

Patterns of photosynthesis and transpiration rates were similar between the artificial input data and empirical data (compare Figures 2, 9A). As the results, the simulated patterns of the expression levels of the transporter genes were similar between them (compare Figures 7, 9B), with local maxima during the day and local minima during the night. The investment efficiency increased by 4.2% at low sensitivity, 10.3% at intermediate sensitivity, and 27.4% at high sensitivity in comparison with that at “constant” setting.

## Discussion

In this study, we proposed a new mathematical model that simulate the Si dynamics in whole plant in rice and investigated the possible mechanisms underlying diurnal variation of the expression level of the transporter genes. To simulate the dynamics of mineral nutrients in rice, we have to simulate not only water flows in the xylem and phloem but also the transport and distribution of mineral nutrients via transporters. Models have been developed that simulate water flows in the xylem and phloem (Daudet et al., 2002; Lacointe and Minchin, 2008; Lobet et al., 2014; Seki et al., 2015) and transport of mineral nutrients from roots or mineral distribution at nodes (Sakurai et al., 2015; Yamaji et al., 2015). However, no study has been conducted on modeling the dynamics of mineral nutrients by considering both water flow and transporter expression level.

The conceptual characteristics of the model proposed here are as follows: (1) it can simulate the dynamics of a mineral nutrient in a whole rice plant while considering plant morphology (multiple leaves, nodes, and stems); (2) the model can simulate mineral transport from roots and its distribution at nodes; and (3) the model can simulate the control of the expression level of the transporter genes in roots. This concept can also be applied to other mineral nutrients and crops if the experimental data on the absorption and distribution of the target mineral nutrients can be obtained.

In the present study, we assumed that three mechanisms control transporter expression levels. The first mechanism is accumulation control, in which a signaling substance is generated in response to Si concentration in leaf cells and is then transported to roots through phloem sap flow. The model based on this mechanism reproduces the experimental data to some extent: the *Lsi1* expression level gradually decreases after root exposure to Si solution of sufficient concentration (Figure 5). The best parameter sets that agree with the empirical data were *dec* = 0.05, *slp*_c_ = 0.01 and *dec* = 0.1, *slp*_c_ = 0.02 (Figure 5). For all parameter sets, the estimated Si concentration in xylem sap was nearly zero during the night because a low transpiration rate at night decreases the differences between water potentials of hydraulic nodes. Interestingly, the concentration of the signaling substance in the leaf xylem showed diurnal variation (Figure 3C) despite the steady increase in the generation rate of the signaling substance in response to the Si concentration in leaf cells (Figure 3B). This phenomenon may be related to the dynamics of phloem sap. At dawn, the difference in the potential between hydraulic nodes in phloem became small (Supplementary Figure 7) because of the depletion of starch in leaves by this time. Therefore, the flow of phloem sap from top to bottom decreased and the signaling substance remained in the leaves. In the root, the concentration of the signaling substance decreased at dawn (Figure 4). The diurnal variation in the transport rate of the signaling substance generated the diurnal variation in the expression level of the transporter genes (Figure 5).

Under the assumption of shortage control, the expression level of the transporter genes gradually decreased, as under the assumption of accumulation control, but local minima were reached at dawn (Figure 6). The downward convex curve of the expression level under shortage control may be attributable to the mechanisms that determine how fast the expression is suppressed. Under accumulation control, the decrease in the suppression rate depends mainly on the rate of generation of the signaling substance in leaves, whereas under shortage control, it depends mainly on the rate of decay of the signaling substance. The convex curve of the expression level under shortage control appears to fit the data reported by Yamaji and Ma (2011). Therefore, shortage control may be a more likely mechanism for the control of Si dynamics than accumulation control from the aspect of the shape of the curve (but see below).

Simulation under the assumption of water stress control shows diurnal variation of the expression level of the transporter genes (Figure 7). Local minima were reached around midnight. All parameter sets produced similar expression cycles, but the amplitudes differed depending on *slp* parameter values. The parameter set of *dec* = 0.2, *slp* = 0.2 fit well the experimental data of Yamaji and Ma (2007), which show a decrease in the *Lsi1* expression level at midnight to one-third of that at daytime.

Why does rice have a control system that generates the diurnal pattern of the expression level of the transporter genes? To answer this question, we compared the investment efficiency at different parameter values. A decrease in the transporter expression level during the night decreased the relative investment into the expression of the transporter genes (Figure 8A). As a result, the investment efficiency was highest in the simulation that had the largest amplitude of the diurnal variation of expression (Figure 8B) because of the difference in the transpiration rates between day and night. During the day, the transpiration rate is high, xylem sap flow is large, and Si absorbed in the root is efficiently transported to the upper tissues. During the night, the transpiration rate is low, xylem sap flow is small, and Si is not efficiently transported. In other words, when a conveyor belt is moving rapidly, many pieces of baggage can be loaded, but loading many pieces of baggage on a slow conveyor belt is not a good strategy. Interestingly, the increasing rate of the Si concentrations in the tissue cells of the roots during the night were higher than during the day (Supplementary Figure 8). It is because “many pieces of baggage” fell from the slow conveyor. As the results, the average Si concentration of all hydraulic nodes constantly increased even during the night to some extent (Supplementary Figure 9). This result is consistent with the previous experimental studies in which the rate of Si uptake did not slow down during the night (Ma and Takahashi, 2002).

We also investigated the investment efficiencies of accumulation control and shortage control. Under accumulation control, the investment efficiencies do not change greatly among parameter sets (Supplementary Figure 10A). Under shortage control, on the other hand, they were greatly decreased at all settings (Supplementary Figure 10B), perhaps because the level of expression of the transporters decreased during daytime rather than nighttime. Therefore, accumulation control may be preferable for rice from the aspect of investment efficiency. Although, it was reported that dehydration stress decreases the expression of Lsi1 and Lsi2 via ABA in root (Yamaji and Ma, 2007), the mechanism that gradually decreases the expression of transporter genes is not well-understood. Evaluating whether accumulation control or shortage control is the actual mechanism in rice is a task for future study.

The transpiration rate during the night used for the present simulation setting (10% of the daytime transpiration rate) may be large from the actual night-time transpiration rate. However, if the actual transpiration rate during the night is lower than 10% (e.g., Nakano et al., 2010), the conclusion discussed above would not change. It is because lower transpiration rate during the night should slow the xylem sap flow.

To confirm the result, we simulated the model with field data and found a similar diurnal pattern of transporter expression (Figure 9). The investment efficiency was highest when the model was simulated with the most sensitive (large-amplitude) parameters (*dec* = 0.2, *slp* = 0.2).

A previous study suggested that the localization and polarity of transporters observed in rice roots provide highest investment efficiency among all possible patterns evaluated (Sakurai et al., 2015). The present study suggests that rice maximizes the investment efficiency in terms of not only the spatial pattern but also the temporal pattern. A gradual decrease in the expression level of Si transporter genes in response to Si concentration in leaf cells might be the mechanism that increases investment efficiency. In rice, many positive effects of Si have been reported with no detectable negative effects of excess Si intake (Ma and Takahashi, 2002). However, the control of the transporter expression level in response to Si concentration in shoot should improve the efficiency of resource allocation.

In the current model, the processes of Si transport in roots and distribution in nodes are simplified. Including more detailed processes will be needed if the aim is to focus on the dynamics of Si at finer scales, such as the dynamics inside and outside of the cell membrane or the localization and polarity of transporters. However, as the current model was designed to describe the dynamics of Si at the whole-plant scale, its degree of simplicity is appropriate. Moreover, the photosynthate dynamics modeled in this study would be a general pattern of plants and does not include characteristic partitioning processes of carbohydrates found in grasses. Grasses store carbohydrates in mainly stem tissue when carbohydrates from the source is greater than whole plant demand (Slewinski, 2012). However, the non-structural carbohydrates in the stem is mainly expended during reproductive growth period (Slewinski, 2012) and leaves would be the main source of carbohydrates during daytime and nighttime in rice (Eom et al., 2012). Therefore, it could be assumed that the downward transport of sucrose is predominant during nighttime at least in the early vegetative period. This was the case of our simulation in the present study. If the model is applied for the Si dynamics during the reproductive period, it might have to be modified so as to include the effect of the storage.

In the current study, the model structure and values of resistance may be oversimplified. The purpose of this study was to propose a new model to investigate qualitatively why rice controls the expression of Si transporter genes. For quantitative understanding of mineral transport, more realistic structure and resistance of water flow values should be reflected in the model, which would be require a large amount of additional experimental data.

## Conclusion

We developed a new model that simulates the dynamics of Si in a whole rice plant by considering Si transport in the roots, its distribution at the nodes, and the control of the expression level of Si transporter genes by a signaling substances. The model reproduced a gradual decrease and diurnal variation of the expression level of the transporter genes observed by Yamaji and Ma (2007, 2011). Our modeling suggests that a considerable reduction in the expression level of Si transporter genes during the night increases investment efficiency (the amount of Si accumulated in top leaf divided by the total expression level of Si transporter genes). Our study suggests that rice has a system that maximizes the investment efficiency for Si uptake in terms of not only the spatial pattern (Sakurai et al., 2015) but also the temporal pattern.

## Author contributions

GS, NY, NM, MY, and JFM designed the study. GS performed the simulations. KO measured field data. All authors contributed to drafting the paper.

### Conflict of interest statement

The authors declare that the research was conducted in the absence of any commercial or financial relationships that could be construed as a potential conflict of interest.

## References

[B1] Cosgrove D. J. (2010). Chapter 3: Water and plant cells, in Plant Physiology, 5th Edn., eds Taiz L. Zeiger E. (Sunderland, MA: Sinauer Associates Inc.), 67–84.

[B2] Daudet F. A. Lacointe A. Gaudillere J. P. Cruiziat P. (2002). Generalized Münch coupling between sugar and water fluxes for modelling carbon allocation as affected by water status. J. Theor. Biol. 214, 481–498. 10.1006/jtbi.2001.2473 11846604

[B3] Eom J. S. Choi S. B. Ward J. M. Jeon J. S. (2012). The mechanism of phloem loading in rice (*Oryza sativa*). Mol. Cells 33, 431–438. 10.1007/s10059-012-0071-9 22453778 PMC3887736

[B4] Epstein E. (1994). The anomaly of silicon in plant biology. Proc. Natl. Acad. Sci. U.S.A. 91, 11–17. 11607449 10.1073/pnas.91.1.11 PMC42876

[B5] Fantke P. Wieland P. Wannaz C. Friedrich R. Jolliet O. (2013). Dynamics of pesticide uptake into plants: from system functioning to parsimonious modeling. Environ. Model. Softw. 40, 316–324. 10.1016/j.envsoft.2012.09.016

[B6] Foster K. J. Miklavcic S. J. (2016). Modeling root zone effects on preferred pathways for the passive transport of ions and water in plant roots. Front. Plant Sci. 7:914. 10.3389/fpls.2016.00914 27446144 PMC4917552

[B7] Fryer M. E. Collins C. D. (2003). Model intercomparison for the uptake of organic chemicals by plant. Environ. Sci. Technol. 37, 1617–1624. 10.1021/es026079k 12731845

[B8] Grieneisen V. A. Scheres B. Hogeweg P. Marée A. F. M. (2012). Morphogengineering roots: comparing mechanisms of morphogen gradient formation. BMC Syst. Biol. 6:37. 10.1186/1752-0509-6-37 22583698 PMC3681314

[B9] Grieneisen V. A. Xu J. Marée A. F. M. Hogeweg P. Scheres B. (2007). Auxin transport is sufficient to generate a maximum and gradient guiding root growth. Nature 449, 1008–1013. 10.1038/nature06215 17960234

[B10] Kuwagata T. Ishikawa-Sakurai J. Hayashi H. Nagasuga K. Fukushi K. Ahamed A.. (2012). Influence of low air humidity and low root temperature on water uptake, growth and aquaporin expression in rice plants. Plant Cell Physiol. 53, 1418–1431. 10.1093/pcp/pcs087 22685088

[B11] Lacointe A. Minchin P. E. H. (2008). Modelling phloem and xylem transport within a complex architecture. Funct. Plant Biol. 35, 772–780. 10.1071/FP08085 32688831

[B12] Landsberg J. Fowkes N. (1978). Water movement through plant roots. Ann. Bot. 42, 493–508. 10.1093/oxfordjournals.aob.a085488

[B13] Lobet G. Pagés L. Draye X. (2014). A modeling approach to determine the importance of dynamic regulation of plant hydraulic conductivities on the water uptake dynamics in the soil-plant-atmosphere system. Ecol. Model. 290, 65–75. 10.1016/j.ecolmodel.2013.11.025

[B14] Ma J. F. Takahashi E. (2002). Soil, Fertilizer, and Plant Silicon Research in Japan. Amsterdam: Elsevier.

[B15] Ma J. F. Yamaji N. Mitani-Ueno N. (2011). Transport of silicon from roots to panicles in plants. Proc. Jpn. Acad. Ser. B 87, 377–385. 10.2183/pjab.87.377 21785256 PMC3171283

[B16] Ma J. F. Tamai K. Yamaji N. Mitani N. Konishi S. Katsuhara M.. (2006). A silicon transporter in rice. Nature 440, 688–691. 10.1038/nature04590 16572174

[B17] Ma J. F. Yamaji N. Mitani N. Tamai K. Konishi S. Fujiwara T.. (2007). An efflux transporter of silicon in rice. Nature 448, 209–213. 10.1038/nature05964 17625566

[B18] Mitani-Ueno N. Yamaji N. Ma F. (2016). High silicon accumulation in the shoot is required for down-regulating the expression of Si transporter genes in rice. Plant Cell Physiol. 57, 2510–2518. 10.1093/pcp/pcw163 27742884

[B19] Nakano S. Kominami Y. Ohno S. Yokoyama K. (2010). Effect of foehn on nighttime sap flow of soybean. J. Agric. Meteorol. 66, 207–216. 10.2480/agrmet.66.4.1

[B20] Sakurai G. Satake A. Yamaji N. Mitani-Ueno N. Yokozawa M. Ma J. F.. (2015). *In silico* simulation modeling reveals the importance of the Casparian strip for efficient silicon uptake in rice roots. Plant Cell Physiol. 56, 631–639. 10.1093/pcp/pcv017 25673476

[B21] Sasaki A. Yamaji N. Ma J. F. (2016). Transporters involved in mineral nutrient uptake in rice. J. Exp. Bot. 67, 3645–3653. 10.1093/jxb/erw060 26931170

[B22] Savant N. K. Snyder G. H. Datnoff L. E. (1997). Silicon management and sustainable rice production. Adv. Agron. 58, 151–199. 10.1016/S0065-2113(08)60255-2

[B23] Seki M. Feugier F. G. Song X. J. Ashikari M. Nakamura H. Ishiyama K.. (2015). A mathematical model of phloem sucrose transport as a new tool for designing rice panicle structure for high grain yield. Plant Cell Physiol. 56, 605–619. 10.1093/pcp/pcu191 25516572

[B24] Slewinski T. L. (2012). Non-structural carbohydrate partitioning in grass stems: a target to increase yield stability, stress tolerance, and biofuel production. J. Exp. Bot. 63, 4647–4670. 10.1093/jxb/ers124 22732107

[B25] Trapp S. (2014). Calibration of a plant uptake model with plant- and site-specific data for uptake of chlorinated organic compounds into radish. Environ. Sci. Technol. 49, 395–402. 10.1021/es503437p 25426767

[B26] Yamaji N. Ma J. F. (2007). Spatial distribution and temporal variation of the rice silicon transporter Lsi1. Plant Physiol. 143, 1306–1313. 10.1104/pp.106.093005 17259286 PMC1820904

[B27] Yamaji N. Ma J. F. (2011). Further characterization of a rice silicon efflux transporter, Lsi2. Soil Sci. Plant Nutri. 57, 259–264. 10.1080/00380768.2011.565480

[B28] Yamaji N. Ma J. F. (2014). The node, a hub for mineral nutrient distribution in graminaceous plants. Trends Plant Sci. 19, 556–563. 10.1016/j.tplants.2014.05.007 24953837

[B29] Yamaji N. Sakurai G. Mitani-Ueno N. Ma J. F. (2015). Orchestration of three transporters and distinct vascular structures in node for intervascular transfer of silicon in rice. Proc. Natl. Acad. Sci. U.S.A. 112, 11401–11406. 10.1073/pnas.1508987112 26283388 PMC4568664

