# Supplementary material for: A Model of Silicon Dynamics in Rice: An Analysis of the Investment Efficiency of Si Transporters
Source: Front Plant Sci. 2017 Jul 11;8:1187. doi: 10.3389/fpls.2017.01187 (PMC5504195; doi:10.3389/fpls.2017.01187)
Supplement: Supplementary file 1 [file Table1.pdf]

Supplementary Material

A model of silicon dynamics in rice:

an analysis of the investment efficiency of Si transporters

Gen Sakurai[1]*, Naoki Yamaji[2], Namiki Mitani-Ueno[2], Masayuki Yokozawa[3], Keisuke Ono[4], and Jian Feng Ma[2]

[1] Statistical Modeling Unit, Division of Informatics and Inventory, Institute for Agro-Environmental Sciences, National Agriculture and Food Research Organization, 3-1-3 Kannondai, Tsukuba 305-8604, Japan

[2] Institute of Plant Science and Resources, Okayama University, 2-20-1 Chuo, Kurashiki 710-0046, Japan

[3] Faculty of Human Sciences, Waseda University, 2-579-15 Mikajima, Tokorozawa 359-1192, Japan

[4] Crop–Climate Interaction Unit, Division of Climate Change, Institute for Agro-Environmental Sciences, National Agriculture and Food Research Organization, 3-1-3 Kannondai, Tsukuba 305-8604, Japan

*** Correspondence:**

Gen Sakurai, Statistical Modeling Unit, Division of Informatics and Inventory, Institute for Agro-Environmental Sciences, National Agriculture and Food Research Organization, 3-1-3 Kannondai, Tsukuba 305-8604, Japan; sakuraigen@affrc.go.jp; +81-29-838-8224

1 Supplementary model description

Here, we explain the model of Daudet et al. (2002) and describe the differences between this model and the model used in our study. We slightly simplified the model of Daudet et al. (2002) and added some equations to stabilize the dynamics of starch and sucrose to increase robustness. We then calibrated some parameters relevant to respiration and ignored fruit development and increase in biomass. In the leaf, the photosynthate is first stored in mesophyll as starch. The changes in starch content was calculated as:

$$\frac{dStarch_i}{dt} = k_{\mathrm{starch}} \cdot V_{\mathrm{sym}(i)}\left(C_{\mathrm{S}(i)\mathrm{Mes}} - c_{\mathrm{targ}}\right)$$

where $Starch_i$ is starch content, k_{starch} and c_{targ} are parameters (all parameters are defined in Supplementary Table 2), $V_{\mathrm{sym}(i)}$ is the volume of the symplast, i is the serial number of the hydraulic node, and $C_{\mathrm{S}(i)\mathrm{Mes}}$ is sucrose concentration in mesophyll (Daudet et al. 2002). We considered that the synthesis and hydrolysis of starch depend on sucrose concentration in leaf mesophyll. Starch is synthesized from sucrose when sucrose concentration is $>c_{\mathrm{targ}}$ and is hydrolyzed to sucrose when

sucrose concentration is $<c_{\mathrm{targ}}$. Sucrose is gradually loaded into the phloem; the flow of sucrose from the mesophyll to the phloem, $J_{\mathrm{S}(i)\mathrm{load}}$, is described by the following equation (Daudet et al. 2002):

$$J_{\mathrm{S}(i)\mathrm{load}} = k_{\mathrm{load}} \cdot Starch_i$$

where k_{load} is a parameter. The respiration rate in the leaf, $R_{\mathrm{m}(i)}$, was calculated as:

$$R_{\mathrm{m}(i)} = \left(k_{\mathrm{res1}} + k_{\mathrm{res2}} \cdot C_{\mathrm{S}(i)\mathrm{Mes}}\right) \cdot Struct_i$$

where k_{res1} and k_{res2} are parameters and $Struct_i$ is structural carbon content. To increase the stability of the model, we added the following equation:

$$\mu_i = \frac{1}{1 + e^{l_1\left(Starch_i - l_2 \cdot V_{\mathrm{cyt}(i)}\right)}}$$

where μ_i regulates the photosynthetic rate according to starch content ($Starch_i$), l_1 and l_2 are parameters, and $V_{\mathrm{cyt}(i)}$ is the volume of the cytoplasm. The photosynthetic rate is multiplied by μ_i: i.e., photosynthesis is suppressed as the leaf starch content increases (Nakano et al. 2000). We arbitrarily chose the values of l_1 and l_2, but assumed that the maximum starch content is ~50 mg g^{-1} dry matter (Okumura and Saka 1989). We also assumed a decrease in the rate of phloem loading with increasing sucrose concentration in the phloem:

$$\gamma_i = \frac{1}{1 + e^{l_3\left(C_{\mathrm{S}(i)} - c_{\mathrm{lim}}\right)}}$$

where γ_i is a regulator of the flow of sucrose from mesophyll to phloem, l_3 and c_{lim} are parameters (value chosen arbitrarily, but the maximum of γ_i is assumed to be ~0.7 mmol mL^{-1}, which is 50% higher than the optimum sucrose concentration in rice; Jensen et al. 2013), and $C_{\mathrm{S}(i)}$ is sucrose concentration in the phloem. Therefore, the equation that describes the flow of sucrose from mesophyll to phloem ($J_{\mathrm{S}(i)\mathrm{load}}$) was changed to:

$$J_{\mathrm{S}(i)\mathrm{load}} = \gamma_i \cdot k_4 \cdot Starch_i$$

where k_4 is a parameter. The maintenance respiration rate was assumed to be proportional to sucrose concentration in the phloem (Daudet et al. 2002):

$$R_{\mathrm{m}(i)} = \left(k_1 + k_2 C_{\mathrm{S}(i)}\right) \cdot Struct_i$$

where $R_{\mathrm{m}(i)}$ is respiration rate, and k_1 and k_2 are parameters.

The starch synthesis rate, $ST_{\mathrm{syn}(i)}$, in tissues other than leaves was described assuming a Michaelis-Menten equation of sucrose concentration (Daudet et al. 2002):

$$ST_{\mathrm{syn}(i)} = V_{\mathrm{cyt}(i)} \cdot \frac{V_{\mathrm{smax}} \cdot C_{\mathrm{S}(i)}}{K_{\mathrm{M}} + C_{\mathrm{S}(i)}}$$

where $V_{cyt(i)}$ is the volume of the cytoplasm, and V_{smax} and K_M are parameters. For simplicity, the local concentrations of sucrose in all tissues other than leaves was considered to be the same as in the phloem. The rate of starch hydrolysis, $ST_{hyd(i)}$, was described as (Daudet et al. 2002):

$$ST_{\mathrm{hyd}(i)} = k_{\mathrm{hyd}} \cdot Starch_i$$

where k_{hyd} is a parameter.

2 Supplementary Tables

Supplementary Table 1. Setting of the simulation.

Explanation	value	Unit
number of leaf	5	
area of leaf (top leaf)	0.0048 (0.00095)	m^2
structural carbon content of leaf (top leaf)	8.1 (1.6)	mmol (s.e.)<br>(sucrose equivalent)
structural carbon content of sheath or stem	3.0	mmol (s.e.)
structural carbon content of node	3.0	mmol (s.e.)
structural carbon content of root	19.0	mmol (s.e.)
xylem flow resistance of leaf (top leaf)	0.064 (0.32)	MPa h mL^{-1}
xylem flow resistance of sheath or stem	0.010	MPa h mL^{-1}
xylem flow resistance of node	0.0050	MPa h mL^{-1}
xylem flow resistance of root	0.17	MPa h mL^{-1}
phloem flow resistance of leaf (top leaf)	2.1 (10.7)	MPa h mL^{-1}
phloem flow resistance of sheath or stem	2.5	MPa h mL^{-1}
phloem flow resistance of node	5	MPa h mL^{-1}
phloem flow resistance of root	16.67	MPa h mL^{-1}
sum of the apoplastic pathway resistance	0.03	MPa h mL^{-1}
tissue volume of cytoplasm of leaf (top leaf)	0.31 (0.062)	ml
tissue volume of cytoplasm of sheath or stem	0.20	ml
tissue volume of cytoplasm of node	0.20	ml
tissue volume of cytoplasm of root	0.08	ml
ratio of the volume of conduit or sieve tube to that of cytoplasm	0.01	

The values are arbitrary decided, but referring to Miyamoto et al. 2001, Matsuo et al, 2009, Daudet et al. 2012, Xiong et al. 2015.

Supplementary Table 2. Symbols and units used in the model.

Symbol	Explanation	Unit
$J_{W(i,j)X}$	axial water flow in xylem from hydraulic node *i* to *j*	mL h^{-1}
$J_{W(i)Lat}$	lateral water flow from xylem to phloem at hydraulic node *i*	mL h^{-1}
$J_{W(i,j)P}$	axial water flow in phloem from hydraulic node *i* to *j*	mL h^{-1}
$J_{S(i,j)}$	axial sucrose flow in phloem from hydraulic node *i* to *j*	mL h^{-1}
$J_{M(i,j)}$	axial Si flow in xylem from hydraulic node *i* to *j*	mL h^{-1}
$J_{S(i)load}$	sucrose flow from mesophyll to phloem at hydraulic node *i*	mmol h^{-1}
$J_{M(i)unload}$	Si flow from xylem to tissue cells at hydraulic node *i*	mmol h^{-1}
$J_{M(o:c)}$	Si flow from external solution (or soil) to root cortex	mmol h^{-1}
$J_{M(c:s)}$	Si flow from root cortex to stele	mmol h^{-1}
$\Psi_{X(i)}$	xylem water potential at hydraulic node *i*	MPa
$\Psi_{P(i)}$	phloem water potential at hydraulic node *i*	MPa
$P_{P(i)}$	phloem hydraulic pressure at hydraulic node *i*	MPa
Π_i	osmotic potential at hydraulic node *i*	MPa
$r_{X(i,j)}$	xylem flow resistance between hydraulic nodes *i* and *j*	MPa h mL^{-1}
$r_{Lat(i)}$	sum of the apoplastic pathway resistance of xylem and phloem at hydraulic node *i*	MPa h mL^{-1}
$r_{P(i,j)}$	phloem flow resistance between hydraulic nodes *i* and *j*	MPa h mL^{-1}
T_i	absolute temperature at hydraulic node *i*	K
$C_{S(i)}$	sucrose concentration in phloem	mmol mL^{-1}
$C_{M(i)}$	Si concentration in hydraulic node *i*	mmol mL^{-1}
$C_{M(i)cyt}$	Si concentration in tissue cells at hydraulic node *i*	mmol mL^{-1}
$C_{M:cor}$	Si concentration in root cortex	mmol mL^{-1}
$C_{M:out}$	Si concentration in external solution or soil	mmol mL^{-1}
$C_{S(i)Mes}$	sucrose concentration in mesophyll at hydraulic node *i*	mmol mL^{-1}
$C_{M(i)DVB}$	Si concentration in DVB at hydraulic node *i*	mmol mL^{-1}

$C_{\mathrm{M}(i)\mathrm{EVB}}$	Si concentration in EVB at hydraulic node *i*	mmol mL^{-1}
$Starch_i$	starch content at hydraulic node *i*	mmol
$V_{\mathrm{sym}\,(i)}$	volume of symplast at hydraulic node *i*	mL
$V_{\mathrm{cyt}(i)}$	tissue volume of cytoplasm at hydraulic node *i*	mL
V_{cor}	tissue volume of cortex	mL
$V_{\mathrm{st}(i)}$	tissue volume of sieve tubes at hydraulic node *i*	mL
$V_{\mathrm{con}(i)}$	tissue volume of conduit at hydraulic node *i*	mL
$Struct_i$	structural carbon content at hydraulic node *i* (sucrose equivalent)	mmol
$R_{\mathrm{m}(i)}$	respiration rate at hydraulic node *i* (sucrose equivalent)	mmol h^{-1}
$ST_{\mathrm{syn}(i)}$	starch synthesis rate at hydraulic node *i* (sucrose equivalent)	mmol h^{-1}
$ST_{\mathrm{hyd}(i)}$	starch hydrolysis rate at hydraulic node *i* (sucrose equivalent)	mmol h^{-1}
μ_i	regulatory factor for photosynthesis	–
γ_i	regulatory factor for sucrose loading	–
α	regulatory factor for transporter expression	–
C_{R}	concentration of signal that regulates the expression of Si transporters	–

Supplementary Table 3. Model parameters.

Symbol	Relevant factor or event	Value	Unit	Reference
R	universal gas constant	0.00831	MPa mL K^{-1}	
k_{starch}	synthesis and hydrolysis of starch	5	h^{-1}	Daudet et al. 2002
k_4	phloem loading of starch	–	h^{-1}	Calibrated
c_{targ}	synthesis and hydrolysis of starch	0.17	mmol mL^{-1}	Daudet et al. 2002
l_1	photosynthesis regulation	50.0	–	Arbitrary
l_2	photosynthesis regulation	0.1	–	Okumura & Saka 1989
l_3	regulation of phloem loading	50.0	–	Arbitrary
c_{lim}	regulation of phloem loading	0.7	mmol mL^{-1}	Jensen et al. 2013
k_1	maintenance respiration	–	h^{-1}	Calibrated
k_2	maintenance respiration	0.0002	mL $mmol^{-1}$	Arbitrary
V_{smax}	starch synthesis	0.1	mL $mmol^{-1}$ h^{-1}	Daudet et al. 2002
K_M	starch synthesis	1	mmol mL^{-1}	Daudet et al. 2002
k_{hyd}	starch hydrolysis	0.5	h^{-1}	Daudet et al. 2002
ρ_i	regulation of concentration in node	different for each node	–	Arbitrary
$k_{M:unload}$	unloading of Si	200	h^{-1} mL^{-1}	Arbitrary
tr_{exo}	activity of transporters located at exodermal Casparian strips	–	mL h^{-1}	Calibrated
tr_{end}	activity of transporters located at endodermal Casparian strips	–	h mL h^{-1}	Calibrated
p_{cm}	permeability of cell membrane	–	mL h^{-1}	Calibrated
slp_c	signal generation in response to Si concentration in tissue cells	–	$mmol^{-1}$ mL	Calibrated
slp_J	signal generation in response to water stress	–	$mmol^{-1}$ h	Calibrated
slp_r	signal generation in response to Si shortage	–	$mmol^{-1}$ h	Calibrated

dec	decay rate of unknown signal	–	–	Calibrated

References

Daudet, F. A., Lacointe, A., Gaudillere, J. P., and Cruiziat, P. (2002). Generalized Münch coupling between sugar and water fluxes for modelling carbon allocation as affected by water status. *J. Theor. Biol.* 214, 481–498. doi:10.1006/jtbi.2001.2473

Jensen, K. H., Savage, J. A., and Holbrook, N. M. (2013) Optimal concentration for sugar transport in plants. *J. R. Soc.* Interface 20130055. http://dx.doi.org/10.1098/rsif.2013.0055

Matsuo, N., Ozawa, K., and Mochizuki, T. (2009) Genotypic differences in root hydraulic conductance of rice (*Oryza sativa* L.) in response to water regimes. *Plant Soil* 316, 25–34.

Miyamoto, N., Steudle, E., Hirasawa, T., and Lafitte, R. (2001) Hydraulic conductivity of rice roots. *J. Exp. Bot.* 52(362), 1835–1846.

Okumura, M., Hirose, T., Hashida, Y., Ohsugi, R., and Aoki, N. (2017) Suppressioin of starch accumulation in 'sugar leaves' of rice affects plant productivity under field conditions. *Plant Prod. Sci.* 20(1), 102–110.

Xiong, D., Yu, T., Zhang, T., Li, Y., Peng, S., and Huang, J. (2015) Leaf hydraulic conductance is coordinated with leaf morphoanatomical traits and nitrogen status in the genus *Oryza*. *J. Exp. Bot.* 66(3), 741–748.
