# Supplementary material for: A Model of Silicon Dynamics in Rice: An Analysis of the Investment Efficiency of Si Transporters
Source: Front Plant Sci. 2017 Jul 11;8:1187. doi: 10.3389/fpls.2017.01187 (PMC5504195; doi:10.3389/fpls.2017.01187)
Supplement: Supplementary file 2 [file Image1.PDF]

Supplementary Material

A model of silicon dynamics in rice:

an analysis of the investment efficiency of Si transporters

Gen Sakurai[1]*, Naoki Yamaji[2], Namiki Mitani-Ueno[2], Masayuki Yokozawa[3], Keisuke Ono[4], and Jian Feng Ma[2]

[1] Statistical Modeling Unit, Division of Informatics and Inventory, Institute for Agro-Environmental Sciences, National Agriculture and Food Research Organization, 3-1-3 Kannondai, Tsukuba 305-8604, Japan

[2] Institute of Plant Science and Resources, Okayama University, 2-20-1 Chuo, Kurashiki 710-0046, Japan

[3] Faculty of Human Sciences, Waseda University, 2-579-15 Mikajima, Tokorozawa 359-1192, Japan

[4] Crop–Climate Interaction Unit, Division of Climate Change, Institute for Agro-Environmental Sciences, National Agriculture and Food Research Organization, 3-1-3 Kannondai, Tsukuba 305-8604, Japan

*** Correspondence:**

Gen Sakurai, Statistical Modeling Unit, Division of Informatics and Inventory, Institute for Agro-Environmental Sciences, National Agriculture and Food Research Organization, 3-1-3 Kannondai, Tsukuba 305-8604, Japan; sakuraigen@affrc.go.jp; +81-29-838-8224

3. Supplementary Figures

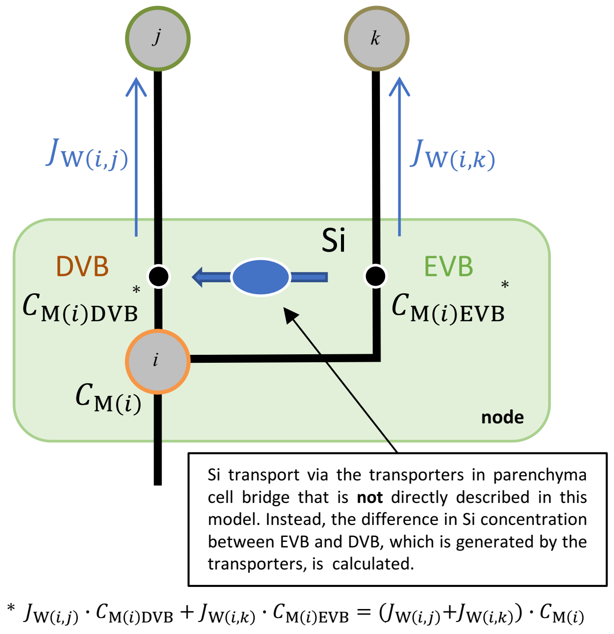

Supplementary Figure 1. Schematic figure of Si transport between EVB and DVB in a node, described in simplified form by Eqs. 15 and 16. $J_{W(i,\,j)}$ and $J_{W(i,\,k)}$ are water flow, $C_{M(i)DVB}$ and $C_{M(i)EVB}$ are Si concentrations in DVB and EVB, and $C_{M(i)}$ Si concentrations in hydraulic node i.

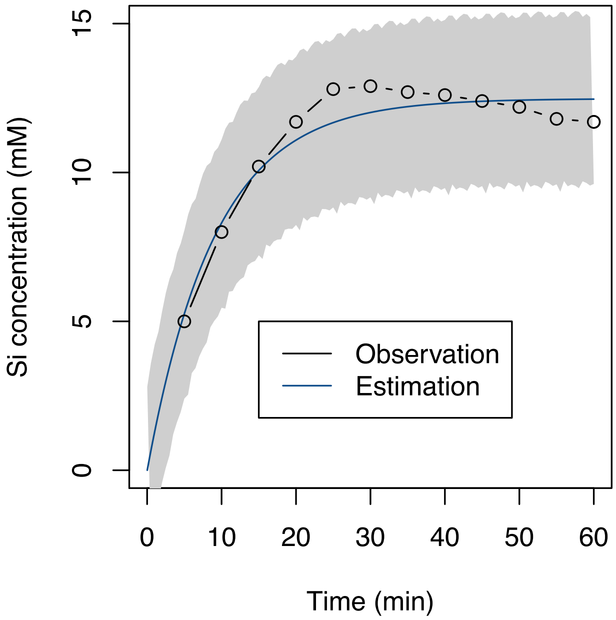

Supplementary Figure 2. Observed and simulated Si concentrations in xylem sap in root. Circles indicate the observed Si concentration and the blue line indicates the estimated Si concentration. Time 0 corresponds to the start of simulation. Grey area indicates the 95% confidence interval.

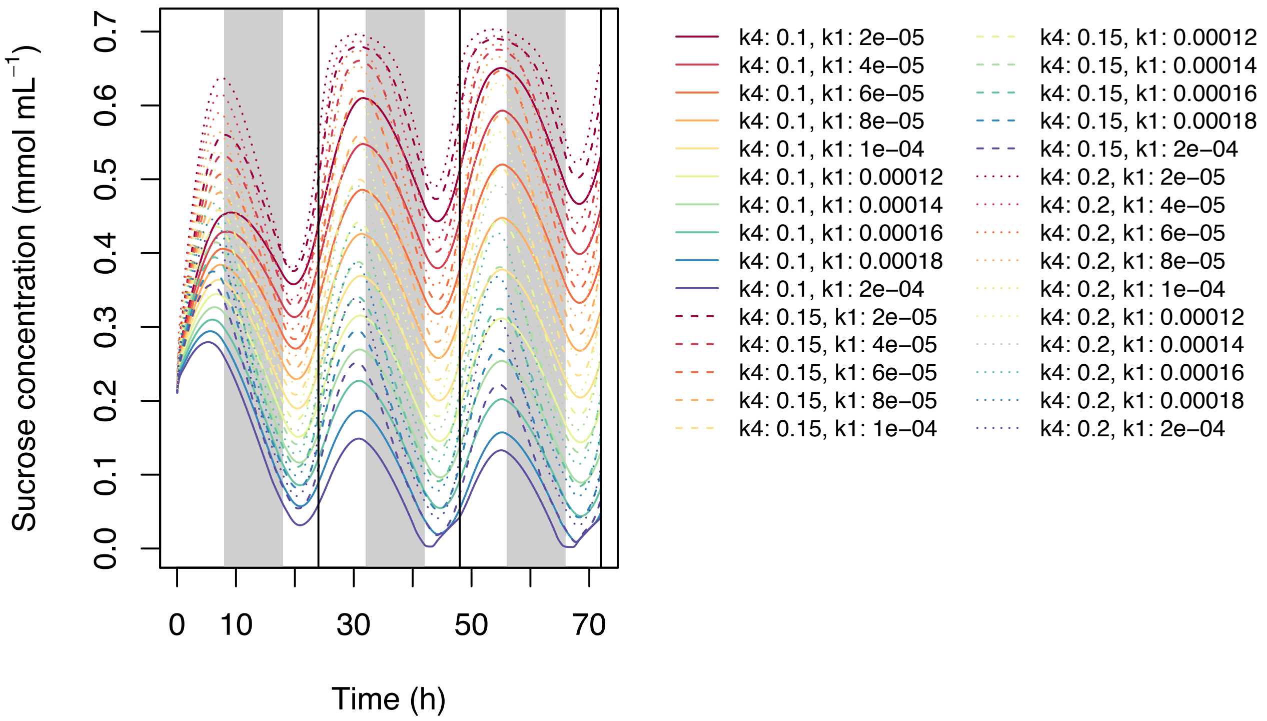

Supplementary Figure 3. Estimated sucrose concentration in the phloem of the top leaf. Time 0 corresponds to the start of simulation. Simulations were performed with multiple parameter settings.

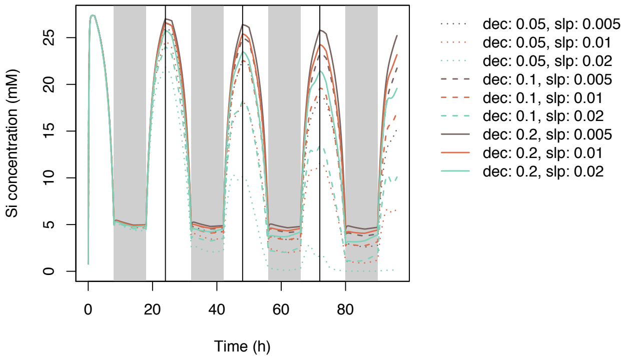

Supplementary Figure 4. Si concentrations in xylem sap of the lowest leaf simulated with multiple parameter sets. Parameter *dec* is the decay rate of the signaling substance. Parameter *slp* is the generation rate of the signaling substance and corresponds to slp_c in Supplementary Table 2.

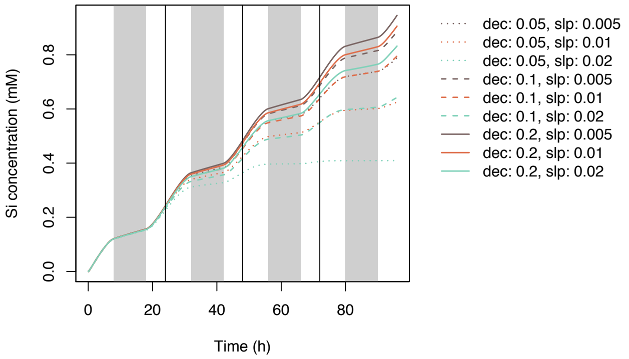

Supplementary Figure 5. Si concentrations in leaf cells of the lowest leaf simulated with multiple parameter sets. Parameter *dec* is the decay rate of the signaling substance. Parameter *slp* is the generation rate of the signaling substance and corresponds to slp_c in Supplementary Table 2.

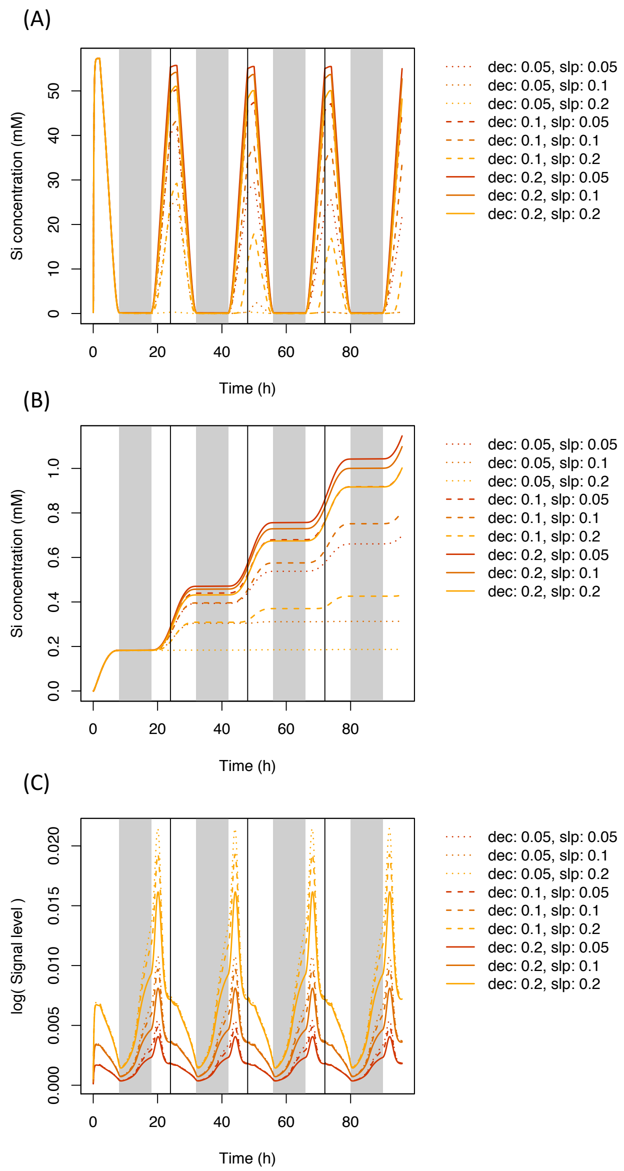

Supplementary Figure 6. Si concentrations in xylem sap (A) and leaf cells (B) of the top leaf and the signal level in xylem sap (C) of the top leaf simulated with multiple parameter sets under the "Water stress control" assumption. Parameter *dec* is the decay rate of the signaling substance. Parameter *slp* is the generation rate of the signaling substance and corresponds to slp_c in Supplementary Table 2.

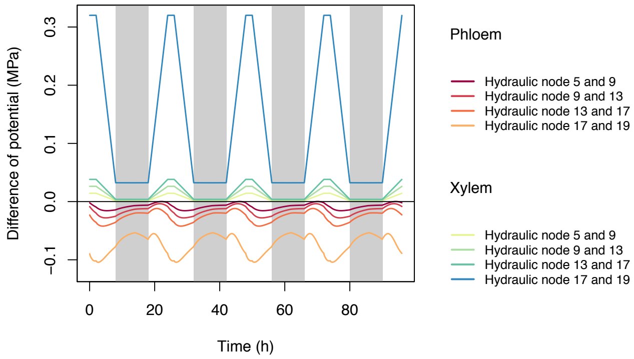

Supplementary Figure 7. Differences in water potential between two hydraulic nodes in the phloem and xylem.

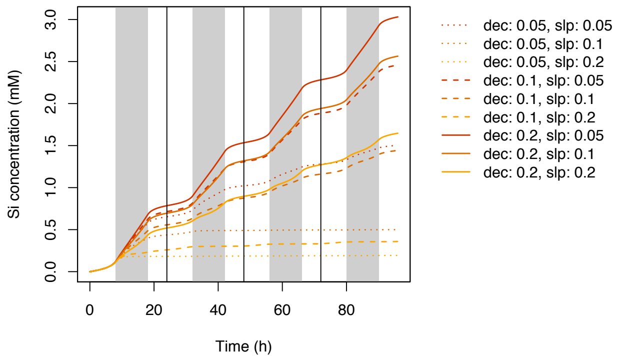

Supplementary Figure 8. Si concentrations in tissue cells of the root simulated with multiple parameter sets. Parameter *dec* is the decay rate of the signaling substance. Parameter *slp* is the generation rate of the signaling substance and corresponds to slp_J in Supplementary Table 2.

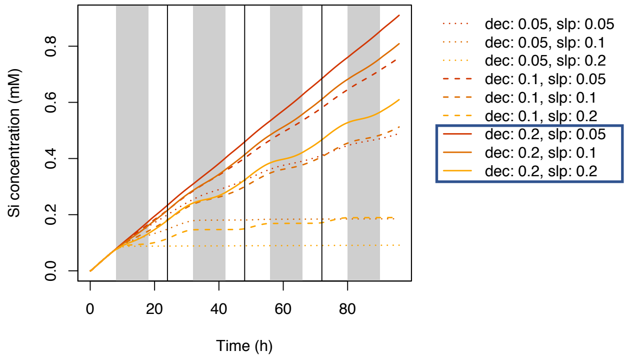

Supplementary Figure 9. Average Si concentrations in tissue cells of all hydraulic nodes simulated with multiple parameter sets. Parameter *dec* is the decay rate of the signaling substance. Parameter *slp* is the generation rate of the signaling substance and corresponds to slp_J in Supplementary Table 2. The parameter sets that is surrounded by blue line are those well fit to the previous experimental data (see Fig. 7).

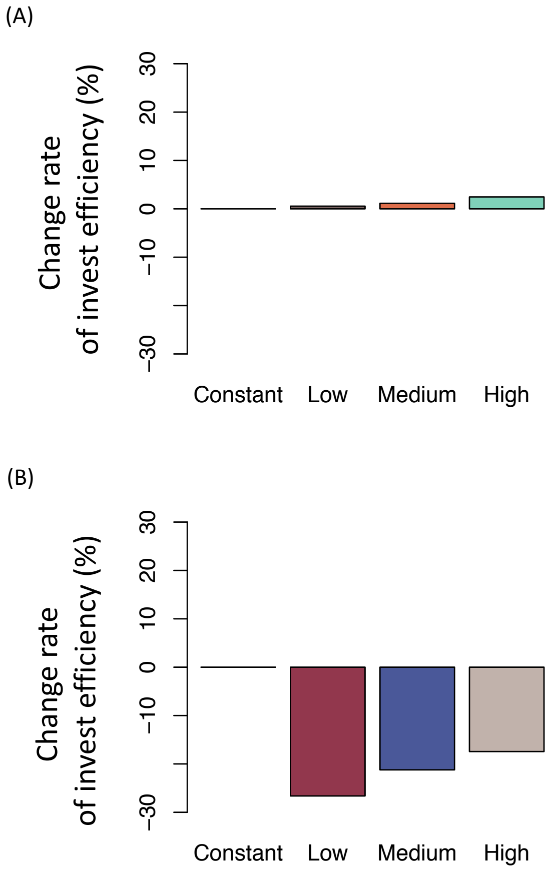

Supplementary Figure 10. Rate of change of investment efficiency relative to that under constant expression of transporter genes under assumptions of (A) accumulation control and (B) shortage control. Under accumulation control, the sensitivity of the parameter set was constant (no sensitivity of Si transporter expression to water stress; *dec* = 0.0, *slp* = 0.0), low (*dec* = 0.2, *slp* = 0.005), intermediate (*dec* = 0.2, *slp* = 0.01), or high (*dec* = 0.2, *slp* = 0.02) (see Fig. 5). Under shortage control, the sensitivity was constant (*dec* = 0.0, *slp* = 0.0), low (*dec* = 0.2, *slp* = 0.01), intermediate (*dec* = 0.2, *slp* = 0.02), or high (*dec* = 0.2, *slp* = 0.04) (see Fig. 6).

frontiers
